# Multiscale EEG network alterations in adolescents with depressive symptoms: static–dynamic connectivity, topological features, and exploratory scalp-level network hubs

**DOI:** 10.3389/fpsyt.2026.1943613

**Published:** 2026-10-26

**Authors:** Linfeng Hou, Mengqi Chen, Jinghui Yang, Yizhong Ren, Ting Peng, Yanru Li

**Affiliations:** School of Physical Education, Shaanxi Normal University, Xi’an, Shaanxi, China

**Keywords:** adaptive threshold; multiscale brain networks, adolescents with depressive symptoms, exploratory neuromodulation hubs, static-dynamic connectivity, topological data analysis

## Abstract

**Background:**

Adolescence is a critical period for brain network development and a vulnerable stage for the emergence of depressive symptoms Although altered brain connectivity has been reported in depression, the multiscale organization of resting-state EEG networks in adolescents with depressive symptoms (ADS) remains insufficiently understood. In particular, conventional network analyses are often constrained by fixed thresholding strategies and single-level observations, which may obscure scale-specific and topology-related abnormalities.

**Methods:**

Eyes-closed resting-state EEG data were collected from 96 adolescents with depressive symptoms and 154 healthy controls. A data-driven adaptive thresholding strategy was used to construct subject-specific brain functional networks. Static functional connectivity, dynamic functional connectivity, and higher-order topological features were analyzed across macro-, meso-, and micro-scale levels. A multidimensional abnormality convergence matrix was further constructed to explore the spatial correspondence between network abnormalities and exploratory scalp-level neuromodulation hubs.

**Results:**

ADS showed scale-dependent abnormalities in brain network organization. At the macro- and meso-scale levels, abnormalities were mainly characterized by theta-band static hyperconnectivity, particularly involving central-region networks, together with reduced dynamic transition flexibility involving prefrontal systems. Micro-scale analysis further localized these alterations to specific connections and nodes. Static connectivity abnormalities were mainly observed along the midline prefrontal–central axis, including Fp1/Fz–FCz connections, whereas dynamic instability was mainly found in right prefrontal–occipital pathways, including Fp2/F4–Oz connections. Higher-order topological analysis indicated vulnerability of right prefrontal nodes, particularly Fp2 and F4. The convergence matrix showed spatial overlap between abnormal network features and the approximate scalp projections of prefrontal neuromodulation sites, while also identifying FCz and Oz as additional nodes that may warrant further investigation.

**Conclusion:**

This study provides a multiscale EEG network characterization of adolescents with depressive symptoms. The findings suggest that ADS-related network alterations may involve coexisting compensatory connectivity changes, reduced dynamic flexibility, and focal topological vulnerability. These results may help refine network-level features and provide hypothesis-generating computational references for future studies exploring individualized neuromodulation strategies in adolescents with depressive symptoms.

## 1 Introduction

Adolescence is a critical sensitive period for the development and remodeling of neural circuits in the brain, and it is also a stage with a high incidence of depression and related mood disorders. (1) Prolonged or severe depressive symptoms not only severely impair adolescents’ executive control functions and cognitive flexibility but also significantly increase the risk of developing major depressive disorder (MDD) and suicide in adulthood. (2) Resting-state electroencephalography (rs-EEG), with its extremely high temporal resolution, has become a vital tool for noninvasively detecting spontaneous rhythmic abnormalities in brain neural networks. (3) However, current EEG-based connectomics research on depression still faces two core translational bottlenecks: First, at the network construction level, how to eliminate volume-conductance effects and avoid topological distortions caused by subjective thresholding; second, at the feature analysis level, how to move beyond traditional single-feature connectivity analyses to characterize high-dimensional spatiotemporal network dynamics and inform future intervention-related research.

In quantifying the connectivity of brain functional networks, the phase lag index provides a highly robust measure for overcoming the volumetric conduction effects inherent in EEG signals by ignoring connections with zero phase difference. However, determining how to sparsify the fully connected matrix in a principled manner remains a key challenge for constructing reliable network topology. Traditional brain network research relies heavily on empirically driven absolute or proportional thresholds. As Adebisi pointed out in a recent network analysis, this fixed-threshold strategy suffers from a critical common flaw in mental disorder research (4): proportional thresholds force all participants’ networks to maintain the same connection density, which essentially obscures the heterogeneity in network connection density caused by pathological factors such as depression; while fixed absolute thresholds are highly prone to causing non-physiological topological fragmentation in the networks of participants with weak baseline connectivity. In particular, when analyzing highly dynamic resting-state EEG data involving rapid shifts in cognitive dFCS, fixed thresholds are completely unable to adapt to the dramatic fluctuations in connectivity baselines over time. Therefore, this study introduces a data-driven adaptive thresholding framework. By tracking time-varying fluctuations in the network baseline and extracting the most significant connections, this framework reduces threshold-related subjective bias and helps preserve subject-specific network topology while attenuating background noise.

While ensuring the fidelity of network reconstruction, the second major translational challenge currently facing connectomics is how to overcome the limitations of traditional, single, static connections and comprehensively deconstruct the high-dimensional spatiotemporal dynamics of the brain networks associated with depression. Traditional brain connectomics research largely relies on static functional connectivity. While this method effectively characterizes robust baseline coupling strengths between electrodes, it tends to treat the brain as a time-invariant linear system, thereby overlooking the transient switching and state evolution of real neural activity on the millisecond timescale. (5) To compensate for this lack of temporal dimension, dynamic functional connectivity state (dFCS) analysis has been widely adopted. dFC analysis is useful for characterizing temporal changes in network organization and may help identify reduced state flexibility in depression-related samples.

It is important to note that both sFC, which characterizes baselines, and dFC, which tracks temporal sequences, are mathematically limited to low-order pairwise interactions (such as node degree and clustering coefficient), and thus remain unable to capture the complex information manifolds and higher-order co-evolutionary patterns of the cerebral cortex. To overcome the analytical blind spots of low-order connectivity, Topological Data Analysis (TDA)—particularly Persistent Homology (PH)—provides a powerful mathematical framework for tracking higher-order geometric features of networks (such as one-dimensional information loops and topological holes) within nonlinear, multiscale spaces. However, traditional PH analysis also has inherent limitations: it often provides only macroscopic, global topological descriptions (such as Betti numbers and the overall persistent landscape), lacking the spatial specificity required to precisely map higher-order geometric vulnerabilities to specific anatomical regions. (6) Addressing this challenge, Mondal et al. recently demonstrated that extending the analytical dimension of TDA to “Node Persistence” enables the precise identification, at an unprecedented microscopic resolution, (7) of focal higher-order hubs that underpin brain network connectivity. These focal higher-order topological parameters may provide exploratory computational information for future studies of scalp-level neuromodulation-related hubs. Combining current clinical guidelines with the latest advances in network-based neuromodulation, candidate targets for non-invasive brain stimulation (NIBS) in depression have gradually expanded from traditional single nodes to multidimensional network hubs. F3 and F4 are commonly used scalp-level approximations of left and right dorsolateral prefrontal cortex stimulation sites in rTMS studies. Meanwhile, with the deepening understanding of the neurobiology of depression, other prefrontal nodes have emerged as promising exploratory targets: for instance, the Fz node (approximating the dorsomedial prefrontal cortex, DMPFC) serves as a critical alternative target for modulating the default mode network (DMN) (8), and the Fp1 and Fp2 nodes located in the frontopolar cortex represent key anatomical anchors for the prefrontal-amygdala reward circuits (9). However, whether these different clinical and exploratory scalp-level targets share the same underlying pathological mechanisms, or whether each exhibits dimensional specificity in brain network impairments, remains poorly understood.

In summary, this study proposes a multiscale brain network analysis framework that integrates adaptive thresholds, static and dynamic functional connectivity, and higher-order topological features, aiming to systematically deconstruct the multidimensional spatiotemporal reorganization patterns in adolescents with depressive symptoms (ADS), ranging from macroscopic global states and mesoscopic local connectivity down to microscopic sensor nodes. To evaluate the potential applications of this data-driven framework in characterizing ADS-associated network alterations and providing exploratory computational references for neuromodulation-related research, this study proposes and tests the following two theoretical hypotheses: (1) Hypothesis 1: Brain network alterations in adolescents with depressive symptoms (ADS) may not represent unidimensional functional decline, but are more likely a complex process intertwining static connectivity, dynamic fluctuations, and local topological distortions. Based on this, we further speculate that such network reorganization may exhibit potential “spatial-scale dependence”: that is, certain key network alterations may only manifest at specific spatial scales, while remaining difficult to detect effectively across other observational dimensions. Therefore, we sought to test whether cross-scale, multi-level observations could reveal specific network features that remain “hidden” at a single spatial scale. (2) Hypothesis 2: Integrating microscopic multidimensional network features can provide computational references for exploring potential neuromodulation hubs. Consequently, we hypothesized that: first, commonly used prefrontal scalp-level stimulation sites may show different patterns of ADS-related network alterations across network feature indices; and second, whole-brain data-driven analysis may help identify additional vulnerable scalp-level nodes beyond traditional empirical sites.

## 2 Materials and methods

### 2.1 Experimental subjects

Participants were screened using a two-stage procedure that combined self-report symptom assessment with professional mental health review. All adolescents completed the Center for Epidemiological Studies Depression Scale (CES-D) and the Patient Health Questionnaire-9 (PHQ-9). Adolescents were assigned to the adolescents with depressive symptoms (ADS) group if they simultaneously met both thresholds: a score of ≥20 on the CES-D and ≥10 on the PHQ-9 (10, 11). Because the CES-D and PHQ-9 are both self-report measures of depressive symptoms and assess closely related constructs, the dual-threshold criterion was used as a conservative screening procedure requiring consistent symptom elevation across two validated scales, rather than as evidence that the two scales captured independent symptom dimensions. Screening results were further reviewed and confirmed by qualified mental health professionals from the school counseling center. A total of 96 adolescents with elevated depressive symptoms and 154 healthy controls (HCs) who scored within the predefined normal range (CES-D< 16 and PHQ-9< 5) were included in the final sample. To reduce potential confounding factors and improve sample comparability, participants in both groups were excluded if they had: (1) a history of alcohol or psychoactive substance misuse; (2) any known central nervous system disorder, severe physical illness, or neurodevelopmental disorder; (3) a history or current use of psychotropic medications (e.g., selective serotonin reuptake inhibitors, SSRIs), thereby reducing potential medication-related confounding effects on EEG oscillatory activity. Furthermore, to specifically target the early symptomatic stage, the ADS group strictly excluded adolescents who had received a formal clinical diagnosis of Major Depressive Disorder or were currently undergoing structured psychiatric treatment. For the HC group, the absence of reported or clinically evident psychiatric disorders was confirmed through professional mental health review. Written informed consent was obtained from all participants and their legal guardians after they had received a full explanation of the study procedures. The study was approved by the Ethics Committee of Shaanxi Normal University (Approval No. 202516012).

### 2.2 EEG signal acquisition and preprocessing

EEG signals were recorded in a dimly lit, quiet, and soundproof room to minimize external environmental interference. Prior to the experiment, participants’ scalps were cleaned to optimize electrode-scalp contact. A 32-channel EEG acquisition system (Brain Vision Recorder, Neuroscan, USA) in conjunction with Curry 8.0 software was used to capture 6 minutes of eyes-closed resting-state EEG data from both the adolescent group with depressive symptoms (ADS) and the healthy control group (HC). The contact impedance between the electrodes and the scalp was strictly maintained below 10 kΩ, and the sampling frequency was set to 1024 Hz. Before the recording commenced, participants were explicitly instructed to remain relaxed and awake, keep their eyes closed, avoid specific cognitive tasks, and minimize swallowing and head or ocular movements to reduce electrooculographic (EOG) and electromyographic (EMG) artifacts. Throughout the session, participants were continuously monitored to ensure data quality and state compliance.

Data preprocessing was performed using the EEGLAB 2013 toolbox in the MATLAB 2022b environment, following these specific steps: First, irrelevant channels (VEOG/HEOG) were removed; Subsequently, the signals were downsampled to 256 Hz, and a 1–30 Hz bandpass filter and a 48–52 Hz notch filter were applied to eliminate power supply noise and other high-frequency interference; Re-referencing was performed using the M1 and M2 electrodes as references; artifacts such as eye movements and electromyography (EMG) were separated and removed using Independent Component Analysis (ICA), with visual/manual confirmation in the EEGLAB interface; segments with signal amplitudes exceeding ±70 μV were rejected; and only clean EEG data with a duration greater than 300 seconds were retained. Finally, to enable high-temporal-resolution dFCS analysis, the continuous artifact-free EEG signals were segmented into non-overlapping 2-s epochs. After preprocessing and segmentation, the retained clean EEG duration, the number of retained 2-s epochs, and the retained-data and rejected-data proportions relative to the original 360-s recording were summarized as quality-control metrics and reported in **Supplementary Table S17**.

### 2.3 Methods for measuring functional connectivity

To comprehensively characterize functional coupling between brain regions and effectively overcome false connectivity estimates that may arise from volume conduction effects in resting-state EEG signals, this study selected the Phase Lag Index (PLI) as the core connectivity metric. Although traditional phase synchronization measures (such as the Phase Locking Value, PLV) are insensitive to changes in signal amplitude, they are highly susceptible to interference from common sources. In contrast, the PLI focuses on the asymmetry of the phase difference distribution—that is, whether a stable leading or lagging relationship exists between EEG signals from two channels. Since spurious synchronization caused by volumetric conduction or a common reference electrode typically manifests as zero phase difference or polarity reversal (phase difference of 0 or π), the PLI assigns a zero weight to such instantaneous phase differences via a sign function, thereby exhibiting high robustness against false-positive connections.

In the actual calculation, the instantaneous phase of each channel’s signal within the target frequency band is first extracted using the Hilbert transform. For two channels x and y, the instantaneous phase difference at time tt was defined as Δϕxy(t)=ϕx(t)−ϕy(t). The PLI was then calculated as follows:


$$PLI=\left|\frac{1}{T}\sum_{t=1}^{T}sign\left[sin\left(\Delta \emptyset_{xy}\left(t\right)\right)\right]\right|$$

Here, *sign* refers to the sign function. Theoretically, the PLI ranges from 0 to 1: a value of 0 indicates no stable phase lead/lag relationship between the two signals (or that the relationship is entirely dominated by volumetric conduction effects); a value of 1 indicates a fully stable, non-zero phase difference lock between the two signals, representing the strongest true functional connectivity.

By performing the above calculations on all non-overlapping 2-s time slices across the four core frequency bands—delta (1–4 Hz), theta (4–7 Hz), alpha (8–13 Hz), and beta (14–30 Hz)—for each adolescent with depressive symptoms (ADS) and healthy control (HC), this study obtained a series of high-temporal-resolution continuous functional connectivity matrices, namely continuous PLI tensors of size 30 × 30. Given the 2-s window length, the approximate number of oscillatory cycles contained in each window was 2–8 cycles for the delta band, 8–14 cycles for the theta band, 16–26 cycles for the alpha band, and 28–60 cycles for the beta band. These high-fidelity matrix data lay a solid foundation for subsequent extraction of subject-specific brain functional networks (BFNs) and for dynamic time-series feature analysis.

### 2.4 Brain functional network construction and adaptive threshold strategies

To improve the reliability of brain network construction using the PLI functional connectivity matrix, this study employed a data-driven adaptive thresholding strategy to sparsify the connectivity matrix. This strategy aims to attenuate spurious connections caused by background noise, residual artifacts, and low-intensity nonspecific couplings, while avoiding network topological biases that may result from fixed absolute thresholds or fixed proportional thresholds. (4) Specifically, this study does not preset a uniform threshold but instead determines individualized thresholds based on the distribution characteristics of each subject’s PLI connectivity matrix to construct subject-specific brain functional networks (BFNs). This method helps preserve the natural differences in connectivity patterns and topological structures across individuals while controlling for noise-induced connections. The specific construction process is as follows:

1. Establishment of a reference baseline network: First, the average PLI matrices for the healthy control (HC) group across all frequency bands were calculated and used as the reference matrix. This matrix represents the expected standard brain connectivity pattern in healthy adolescents, providing a robust baseline for subsequent quantification of abnormal connectivity in adolescents with depression.
2. Identification of Most Significant Connections (MSCs): Within a brain network, nodes contribute differently to network properties. This study adopted the concept of “Most Significant Connections (MSCs)” to capture topological edges that undergo substantial changes between the depressed state and the healthy reference state. Specifically, based on the Eigenvector Centrality metric—a feature vector of network nodes—the most influential core electrode nodes in the network were identified. Subsequently, all edges connecting these core nodes to one another, as well as those connecting core nodes to non-core nodes, were collectively defined as the set of MSCs.
3. Calculation of Adaptive Thresholds: To filter out noise while ensuring network connectivity, personalized adaptive thresholds were calculated for each frequency band of every participant based on the extracted MSCs. These thresholds were determined jointly by the connection strength of the participant’s own MSCs and that of the MSCs in the healthy reference group. The calculation formula is as follows:

$$Threshold=\frac{\left(PLI_{sub}^{MSCs}\right)+\left(PLI_{ref}^{MSCs}\right)}{2}$$

Here, $\left(PLI_{sub}^{MSCs}\right)$ represents the average PLI value of the target subject within the MSCs set, while $\left(PLI_{sub}^{MSCs}\right)$ represents the average PLI value of the healthy reference group within the same MSCs set. The advantage of this formula is that it performs a relative standardization calibration of the subject’s individual network characteristics against the healthy baseline network.

4. Brain Functional Network (BFN) Generation: Finally, the aforementioned adaptive threshold is applied to the subject’s raw continuous PLI matrix. If the connection strength between a pair of nodes is greater than or equal to this threshold, the edge is retained; otherwise, it is filtered out. At the same time, to prevent network fragmentation, the algorithm forces the retention of all MSCs during the thresholding process to maintain the network’s core connectivity.
5. To verify the neurobiological topological validity of the thresholded brain functional network (BFN)—as opposed to a purely random structure driven by noise—we conducted a non-randomness test of its small-world properties. Specifically, for each extracted BFN, we calculated its empirical clustering coefficient C and characteristic path length L, and generated equivalent random networks and rule-based lattice networks with the same number of nodes, edges, and degree distribution as null models. Let the clustering coefficient and path length of the random network be denoted as C_r and L_r, respectively, and those of the lattice network as C_l and L_l, respectively. To overcome the limitation of traditional single-benchmark small-world metrics being susceptible to network scale, we adopted the dual-reference normalized metrics SW1 and SW2 proposed by Adebisi et al. for a comprehensive evaluation:

The SW1 metric quantifies the degree of deviation of a network relative to both random and lattice networks, and is calculated as follows:


$$\text{sw}=\frac{{\text{L}}_{\text{r}}}{\text{L}}-\frac{\text{C}}{{\text{C}}_{\text{l}}}$$

$$\text{S}{\text{W}}_{1}=1-\left|\text{sw}\right|$$

The SW2 metric further combines the normalized relative positions of path length and the clustering coefficient, providing a more comprehensive assessment of small-world properties:


$$\text{S}{\text{W}}_{2}=\frac{\text{L}-{\text{L}}_{\text{l}}}{{\text{L}}_{\text{r}}-{\text{L}}_{\text{l}}}\times \frac{\text{C}-{\text{C}}_{\text{r}}}{{\text{C}}_{\text{l}}-{\text{C}}_{\text{r}}}$$

Given that this study employed a continuous network analysis strategy of 2 seconds per slice, the dFCS of the brain network may exhibit transient fluctuations across different slices. To obtain robust subject-level topological validation results, this study extracted the median values of SW1 and SW2 for each subject across all valid time slices. Only when the generated network consistently exhibited a small-world coefficient significantly greater than 0 was it determined that this data-driven adaptive threshold successfully preserved the brain’s complex network integration and segregation characteristics, thereby demonstrating that the constructed subject-specific brain network possessed the statistical validity required for subsequent quantitative analysis and feature extraction.

6. To ensure methodological fairness and rule out potential topological biases driven by unequal network sparsities, the average applied threshold and the resulting network density (defined as the absolute number of retained edges) were calculated for each subject across all valid time slices to assess intergroup consistency. This step was designed to empirically verify that any observed topological alterations stem from strictly comparable network backbones rather than systematic algorithmic artifacts.

### 2.5 Cross-scale feature extraction from brain networks

Traditional neuroimaging analyses are often limited to a single spatial scale or a static temporal dimension, making it difficult to comprehensively deconstruct the spatiotemporal evolution and high-dimensional geometric distortions exhibited by the brain during complex emotional regulation. To address this limitation, this study built upon an adaptive-threshold brain network framework and integrated static functional connectivity, dynamic functional connectivity, and persistent-homology-based topological data analysis (TDA) to construct a cross-scale, multidimensional analytical framework spanning the macro-, meso-, and micro-levels. Within the mesoscale component of this framework, the 30 electrodes were assigned to five anatomical regions for regional-level network analysis: the frontal region included Fp1, Fp2, F7, F3, Fz, F4, and F8; the central region included FC3, FCz, FC4, C3, Cz, C4, CPz, CP3, and CP4; the temporal region included FT7, FT8, T7, T8, TP7, and TP8; the parietal region included P7, P3, Pz, P4, and P8; and the occipital region included O1, Oz, and O2. Boundary electrodes were assigned according to their dominant anatomical proximity and their role in the regional sensor-level network. FC3/FCz/FC4 and CP3/CPz/CP4 were grouped into the central region to capture the frontocentral–central–centroparietal peri-rolandic sensorimotor axis, whereas FT7 and FT8 were assigned to the temporal region because of their lateral frontotemporal location. This electrode-to-region assignment was applied consistently across mesoscale static connectivity, dynamic connectivity, and region-averaged node persistence analyses.

#### 2.5.1 Macroscopic network characteristics

At the macro level, this study aims to quantify the steady-state clustering strength, state-switching resilience, and overall complexity of the brain’s resting-state network in maintaining high-dimensional information topologies across the entire brain.

1. Global Static Connectivity (Global sFC): Measures the average synchronization strength of the brain network across the entire time series. It is defined as the global mean of all off-diagonal edges in the subject’s whole-brain 30×30 connectivity matrix across the entire time domain.
2. Global Dynamic Functional Connectivity States (dFCS): To capture the macroscopic resilience of whole-brain dynamic states, this study introduces an unsupervised machine learning clustering approach (12). First, principal component analysis (PCA, retaining 90% cumulative variance) was used to reduce the dimensionality of the whole-brain connectivity vectors. Subsequently, the K-Means clustering algorithm was applied to the globally concatenated dataset (encompassing all subjects and all valid time windows simultaneously) to identify representative whole-brain dynamic functional connectivity states (dFCS).

To ensure the robustness of the dFCS solutions, the algorithm was executed with 15 independent random initializations. To determine the optimal number of states (k), an independent quantitative evaluation was conducted for k values ranging from 2 to 8. This evaluation utilized the Silhouette Score to assess cluster cohesion and separation, and the Elbow Method (Sum of Squared Errors, SSE) to track the reduction in within-cluster variance. Because purely distance-based mathematical metrics in high-dimensional neuroimaging data often favor oversimplified binary partitions, our final selection criterion for k strictly required a balance between algorithmic metrics (seeking an elbow point of diminishing returns in SSE) and neurobiological interpretability. The goal was to avoid the limited neurobiological interpretability of a two-state solution while preserving sufficient state resolution to capture multi-stable network transitions associated with depressive symptoms. Finally, by calculating the fractional time spent in different dFCS, the total number of transitions, and the Markov transition probabilities, we quantified the temporal flexibility of whole-brain dFCS transitions.

(3) Global Topological Manifold (Global TDA): The thresholded PLI matrix is mapped to a nonlinear metric space distance matrix $D_{ij}\left(t\right)=\sqrt{2\left(1-A_{ij}\left(t\right)\right)}$. By constructing a Vietoris-Rips simplicial complex, we extract the H1-dimensional global persistent entropy $\left(E=-\sum P_{i}\ln P_{i}\right)$ and the *L*^1^ and *L*^2^ norms of the first-level persistent landscape function, to characterize the lifespan diversity and geometric “volume” of higher-order topological circuits across the entire brain.

Building upon the baseline evaluation of network density, a reference-independent sensitivity analysis was conducted to further verify the robustness of the macroscopic findings and rule out potential algorithmic artifacts induced by the HC-referenced thresholding. We reconstructed the networks for both groups by forcing the retention of the top 15%, 20%, and 25% of the strongest connections to ensure completely identical topological sparsity, and repeated all statistical comparisons of the macroscopic metrics under these strictly controlled densities.

#### 2.5.2 Brain region characteristics at the mesoscale

To evaluate the topological capacity and interactive synergy of large-scale anatomical brain regions, this study precisely mapped 30 electrodes to five classical brain regions (frontal, central, parietal, occipital, and temporal lobes):

1. Mesoscale static connectivity (Meso-sFC): For any two brain regions A and B (or within the same brain region), the connectivity strength is defined as the average weight across the entire time domain of all node pairs belonging to that combination.
2. Meso-dynamic fluctuations (Meso-dFC): Quantifies the transient instability of specific brain region pathways during dFCS transitions, defined as the ratio of the standard deviation of connection strength to the mean over time, i.e., the temporal coefficient of variation (Temporal CV = SD/Mean). To rule out the potential confounding effect of baseline mean connectivity on this CV metric, the absolute temporal standard deviation (Absolute SD) without mean normalization was additionally extracted as a supplementary validation. Statistically, this validation was explicitly designed as a targeted *post-hoc* analysis restricted only to pathways exhibiting significant primary CV alterations, thereby avoiding redundant global multiple comparisons.
3. Meso-topological carrying capacity (Meso-NP): This metric was calculated as the mean normalized node persistence across electrodes within each region to assess the regional distribution of higher-order topological alterations.

#### 2.5.3 Micro-scale edge and node characteristics

Guided by anomalies in macroscopic and mesoscopic properties, this study delves further into the specific microscopic electrode scale to examine sensor-level edge and node features that may underlie the macroscopic and mesoscopic network alterations.

To characterize edge-level connectivity features underlying the macro- and meso-scale findings, we extracted pairwise connectivity measures from the 30 × 30 whole-brain sensor array. Static connectivity strength (sFC) was calculated as the average PLI across the full recording period. Dynamic connectivity variability (dFC), defined as the coefficient of variation (standard deviation/mean) of each edge across time windows, was used to quantify temporal variability in edge-wise connectivity. Consistent with the mesoscale analysis, Absolute SD validation was also applied to the microscopic connections.

Edge-level static and dynamic connectivity features may be accompanied by changes in higher-order topological organization. To characterize node-level topological features, we extracted two persistent-homology-derived indices for each electrode: node frequency (NF) and normalized node persistence (NP). Node frequency (NF) was defined as the number of distinct H_1_ topological loops in which a given electrode node participated, reflecting how frequently that node contributed to higher-order network loops. Normalized node persistence (NP) quantified the total lifetime of H_1_ loops involving that node, normalized by the global topological filtration range, thereby reflecting the persistence of that node’s participation in higher-order topological structures. The calculation formula is as follows:


$$NP\left(v\right)=\frac{\sum_{i\in S_{v}}\left(d_{i}-b_{i}\right)}{max_{j}d_{j}-min_{j}b_{j}}$$

where *S_v_* is the set of all one-dimensional topological loops in which node *v* participates.

### 2.6 Spatial mapping of multidimensional anomalous features and exploratory scalp-level network hubs

To examine whether network-derived microscopic nodal abnormalities spatially overlapped with exploratory scalp-level neuromodulation hubs, we constructed a sensor-level multidimensional convergence matrix.

In clinical psychopathology practice, non-invasive brain stimulation (NIBS), particularly repetitive transcranial magnetic stimulation (rTMS) targeting the prefrontal cortex, is a crucial physical intervention for addressing executive control dysfunction in adolescents with depression. To explore the spatial correspondence between computational network features and potential neuromodulation sites, this study evaluated five key prefrontal nodes. It must be strictly noted that these 10–20 EEG system coordinates represent approximate scalp-level projections rather than precise point-to-point cortical targets. Within this set, F3 and F4 serve as scalp-level approximations for the established classical clinical targets, whereas Fz, Fp1, and Fp2 are included as promising exploratory network hubs. Their corresponding set of scalp mapping coordinates is defined as:


$$\Omega_{\text{NIBS}}=\left\{\text{F}3,\text{F}4,\text{Fp}1,\text{Fp}2,\text{Fz}\right\}$$

To summarize edge-level connectivity alterations at the scalp-electrode level, we converted microscopic edge-wise static and dynamic connectivity measures into nodal metrics. For each electrode i, nodal static connectivity strength (Nodal sFC) was calculated as the mean static connectivity between electrode i and all other electrodes k (k ≠ i; total number of electrodes, N = 30). Similarly, nodal dynamic connectivity variability (Nodal dFC) was calculated as the mean temporal variability of dynamic connectivity between electrode i and all other electrodes. This edge-to-node aggregation allowed microscopic connectivity abnormalities to be summarized at the scalp-electrode level for subsequent multidimensional convergence mapping. The calculation formulas are as follows:


$$sFC_{i}=\frac{1}{N-1}\sum_{k\neq i}\omega_{i,k}$$

$$dFC_{i}=\frac{1}{N-1}\sum_{k\neq i}cv_{i,k}$$

In the equation, *ω*_i,k_ and cv_i,k_ represent the static functional connectivity weight and the dynamic temporal coefficient of variation between electrodes i and k, respectively.

Building on this foundation, this study overcomes the limitations of traditional approaches that examine only the overlap of a single topological metric. By integrating the aforementioned dimension-reduced nodal features derived from static and dynamic connectivity (sFC and dFC) with the two related persistent-homology-derived topological indices (NP and NF), we constructed a whole-brain, sensor-level “Multi-dimensional Pathological Convergence Matrix.” This matrix was designed to quantitatively assess the extent to which specific scalp-level nodes show convergent abnormalities across static connectivity, dynamic instability, and higher-order topological features.

Building upon the baseline evaluation of network density and the macroscopic sensitivity analyses, a bootstrap stability test was conducted to validate the rationality of the microscopic findings and the exploratory scalp-level hub mapping. We resampled the continuous PLI tensors across time slices with replacement for 1,000 iterations to repeatedly recalculate the Eigenvector Centrality and extract the Most Significant Connections (MSCs). The stability of these core nodes and connections was quantified using the Jaccard index overlap rate across iterations, thereby providing a stability estimate for the microscopic node analysis and exploratory scalp-level hub mapping.

### 2.7 Statistical analysis methods

All data processing and statistical analyses were performed in a Python 3.9 environment using SciPy and Statsmodels. Normality and homogeneity of variance were assessed using the Shapiro-Wilk and Levene tests, respectively. For between-group comparisons between the healthy control (HC) and adolescents with depressive symptoms (ADS) groups, continuous variables were analyzed using a two-sided independent-samples t-test when they were normally distributed with homogeneous variances, Welch’s t-test when they were normally distributed but showed unequal variances, or the nonparametric Mann-Whitney U test when they showed skewed, non-normal, or threshold-defined distributions. Categorical variables were compared using the chi-square test. To control for multiple comparisons in EEG network features, all initial p-values—except for demographic and baseline clinical comparisons—were corrected using the false discovery rate (FDR). The final statistical significance level was set at a corrected PFDR< 0.05. Features meeting this threshold were annotated in the visualizations with * (*P*_FDR_< 0.05), ** (*P*_FDR_< 0.01), and *** (*P*_FDR_< 0.001).Across the entire multiscale framework, a total of 2,284 feature-level statistical comparisons were performed (4 macro-level tests, 60 meso-level tests, 1,740 micro-edge tests, and 480 micro-node tests). At an uncorrected *α* = 0.05 level, approximately 114 false positives would be expected purely by chance. To address the risk of false positives in high-dimensional testing, all initially significant findings were further subjected to a family-wise non-parametric permutation test (100,000 iterations) strictly preserving the original family sizes, alongside a 5,000-iteration Bootstrapping procedure to derive the 95% confidence intervals (95% CIs) of the effect sizes. Because node frequency (NF) and node persistence (NP) were both derived from the same H_1_ persistent-homology computation, an additional *post-hoc* dependence analysis was conducted to evaluate their statistical non-independence. For node-level features showing concurrent NF and NP significance, Spearman correlations were calculated across participants between subject-level NF and NP values within the corresponding frequency band. This analysis was used only to guide the interpretation of concurrent topological findings and was not treated as an independent discovery analysis.

## 3 Results

### 3.1 Clinical and demographic characteristics

**Table 1** presents the demographic characteristics and clinical scale scores of the adolescents with depressive symptoms (ADS) group and the healthy control (HC) group. No statistically significant between-group differences were observed in age (ADS: 15.8 ± 1.4 years; HC: 15.5 ± 1.6 years; independent-samples t-test, t = 1.750, p = 0.081) or sex distribution (ADS: 50 males/46 females; HC: 79 males/75 females; chi-square test, χ^2^ = 2.560, p = 0.110), indicating that the two groups were comparable in basic demographic characteristics.

**Table 1:** Comparison of demographic and baseline characteristics between adolescents in the group with depressive symptoms and the healthy control group (HC).

Indicators	ADS(N=96)	HC(N=154)	*Statistic*	*p*
Age	15.8 ± 1.4	15.5 ± 1.6	t=1.750	0.081
Gender (Male/Female)	50/46	79/75	χ^2^ = 2.560	0.110
CES-D	22.5 ± 2.2	8.3 ± 3.1	*U* = 14784	*p* < 0.001
PHQ-9	11.5 ± 1.6	2.1 ± 1.3	*U* = 14784	*p* < 0.001

Data are presented as mean ± SD, except for sex, which is presented as male/female counts. Age was compared using an independent-samples t-test, sex distribution was compared using the chi-square test, and CES-D and PHQ-9 scores were compared using Mann–Whitney *U* tests. For CES-D and PHQ-9, *U* values are reported for ADS relative to HC. Because the ADS and HC groups were defined using non-overlapping symptom-scale thresholds, all ADS scores were higher than all HC scores for both scales; therefore, *U* reached the maximum possible value of 14,784 for both comparisons. CES-D, Center for Epidemiological Studies Depression Scale; PHQ-9, Patient Health Questionnaire-9; ADS, adolescents with depressive symptoms; HC, healthy controls.

Regarding clinical baseline characteristics, CES-D and PHQ-9 scores were significantly higher in the ADS group than in the HC group. Because these symptom scores were scale-based and threshold-defined screening variables, group differences were assessed using Mann–Whitney U tests rather than parametric t-tests. The ADS group showed higher CES-D scores than the HC group (ADS: 22.5 ± 2.2; HC: 8.3 ± 3.1; U = 14,784, p< 0.001) and higher PHQ-9 scores than the HC group (ADS: 11.5 ± 1.6; HC: 2.1 ± 1.3; U = 14,784, p< 0.001). These results confirm the expected separation between the ADS and HC groups based on the predefined symptom-screening criteria.

### 3.2 Validation of the adaptive thresholding strategy and network construction results.

Constructed continuous-time brain functional networks (BFNs) can effectively suppress background noise and spurious connections while maximizing the fidelity of intrinsic neural coupling patterns. This study first examined the temporal consistency of the adaptive thresholding framework across four classic frequency bands for representative healthy control (HC) and adolescents with depressive symptoms (ADS) subjects, as shown in **Figure 1**. The results show that, over the entire scanning cycle, the connection density of the continuous brain functional network (BFN) in both groups of participants exhibited distinct time-varying nonlinear fluctuations. Correspondingly, the adaptive threshold trajectories exhibited continuous oscillations along the time axis but overall converged within narrow intervals specific to each frequency band. Specifically, the threshold fluctuations in the low-frequency bands had a wider range (e.g., the Delta band exhibited high-amplitude oscillations primarily between 0.15 and 0.5, while the Alpha band fluctuated between 0.2 and 0.5), whereas the thresholds in the high-frequency bands generally converged to a narrow range at lower values (the Theta band was concentrated between 0.15 and 0.35, while the Beta band is strictly confined to the range of 0.1 to 0.25).

**Figure 1:**
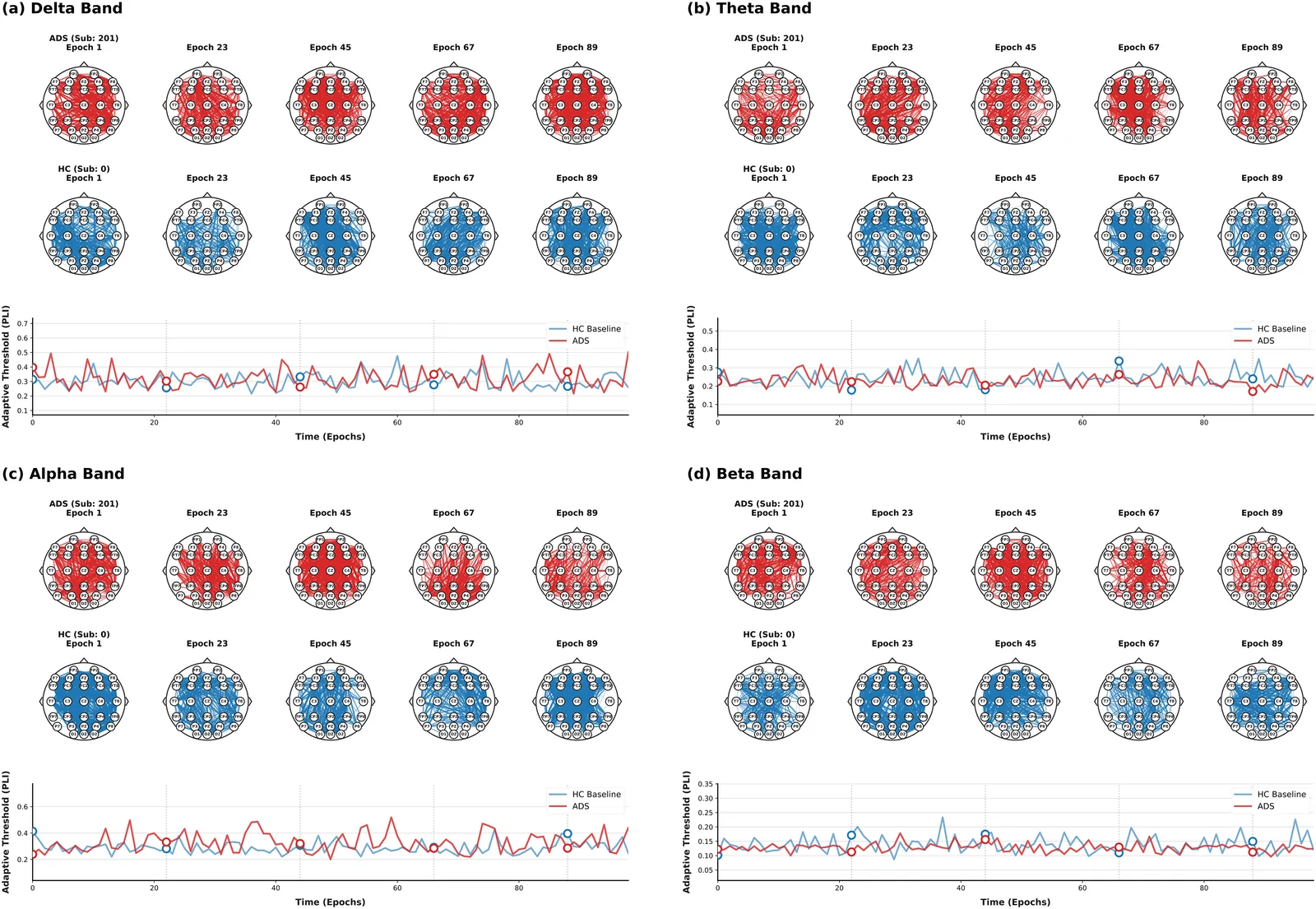
Examples of the temporal evolution of continuous brain functional networks and adaptive threshold trajectories for sample participants. Panels **(a–d)** show the dynamic network characteristics of sample participants in the HC group (blue, Sub: 0) and the ADS group (red, Sub: 201) across four classic frequency bands, respectively. The upper part of each panel shows the spatial topological evolution of the continuous functional brain network across five representative time windows; the lower part shows the time-varying PLI thresholds used for adaptive network sparsification. This figure visually validates the temporal consistency of the adaptive thresholding framework.

Further quantitative evaluation of the generative networks’ physiological topological properties is illustrated by the box plots and underlying scatter plots in **Figure 2**, which visually demonstrate the statistical characteristics of the dual small-world coefficients (SW1 and SW2) across the two groups. Quantitative statistical data show that, across all four frequency bands, the generated networks of both the HC group and the ADS group robustly satisfy the mathematical criteria for a non-random small-world architecture. Specifically, for the SW1 metric—based on the ratio of the clustering coefficient to the characteristic path length—the group-specific medians consistently converge to values near 0.94–0.95 across all four frequency bands, with the vast majority of samples clustered tightly within the core high-density range of 0.88 to 0.96; Meanwhile, for the SW2 metric used as supplementary validation, the overall data range was between 0.6 and 1.4, and the medians across each frequency band were stably anchored near the theoretical benchmark thresholds of 0.9 to 1.1 (e.g., the median in the Alpha band was close to 1.1, while the medians in the Theta and Beta bands are close to 0.9). These quantitative results objectively demonstrate that, in both the HC and ADS groups, the continuous network sequences—after adaptive thresholding—maintain extremely stable, non-random small-world geometric properties across all frequency bands.

**Figure 2:**
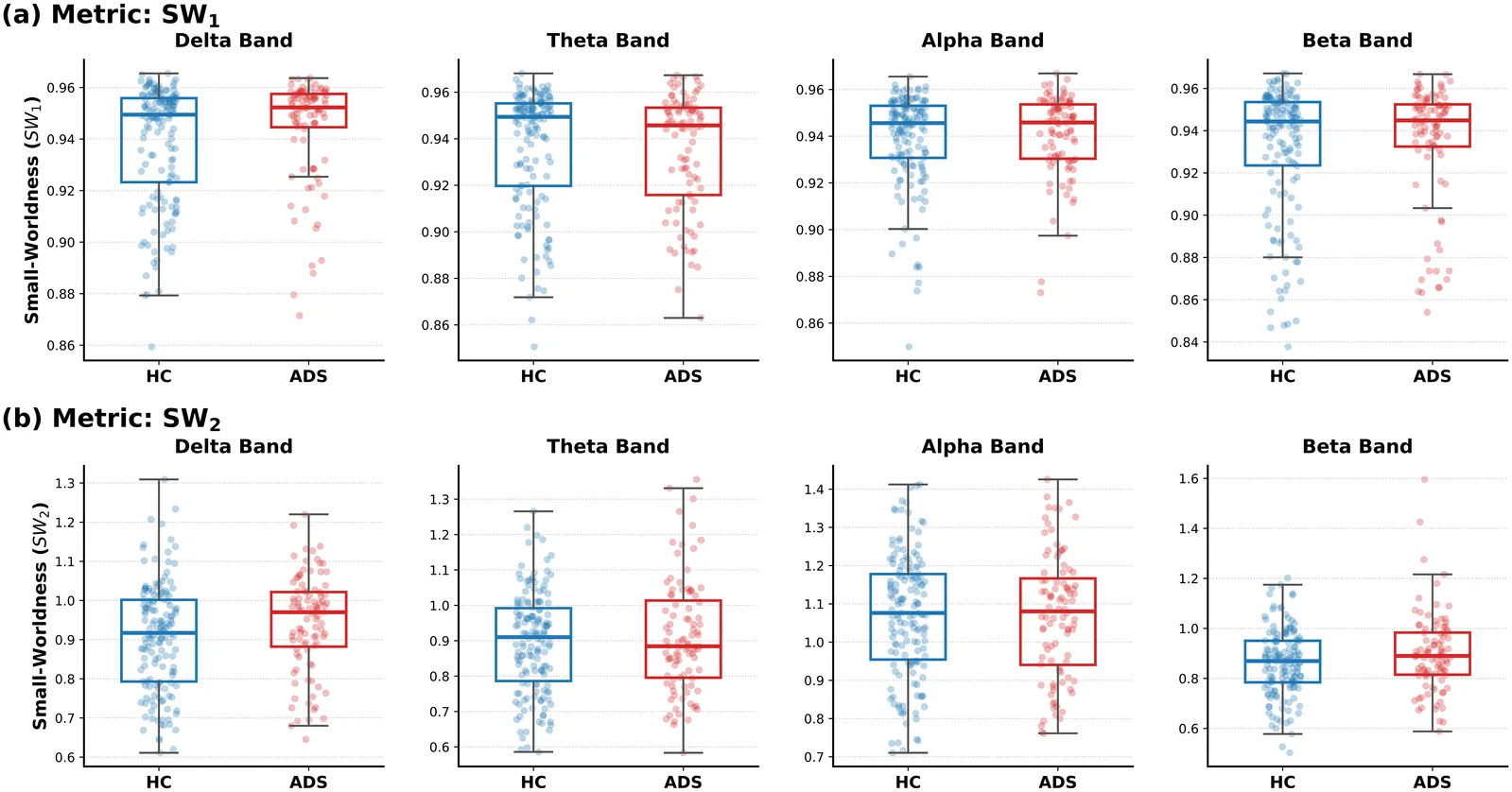
Topological quantification assessment of the constructed functional brain network: Global validation of dual small-world properties. Panels **(a, b)** show the distribution of small-world coefficients based on dual metrics (SW1 and SW2) across the four frequency bands, respectively, aiming to validate the authenticity and non-randomness of the brain functional networks (formulated BFNs) constructed via adaptive thresholding. These quantitative results regarding topological dimensions confirm the authenticity of the constructed networks, laying a reliable data foundation for subsequent cross-scale analyses of pathological network dynamics.

As detailed in **Table 2**, the ADS group exhibited a significantly higher average threshold compared to the HC group exclusively in the Theta band, with no significant differences observed in the remaining frequency bands. Crucially, the average network densities (retained edges) across all four frequency bands showed no significant intergroup differences.

**Table 2:** Statistical comparison of adaptive thresholds and retained edges between HC and ADS groups.

Frequency band	Metric	HC (n=154)	ADS (n=96)	*U*	*r*	*P*	*P_FDR_​*	*Sig.*
Delta	Avg Threshold	0.301 ± 0.015	0.299 ± 0.016	6660.5	-0.083	0.189	0.302	Ns
	Avg Edges	219.26 ± 8.90	221.15 ± 8.40	8383.5	0.113	0.075	0.149	Ns
Theta	Avg Threshold	0.242 ± 0.011	0.256 ± 0.036	9554.5	0.246	<0.001	<0.001	***
	Avg Edges	216.85 ± 10.38	215.73 ± 13.38	6887.5	-0.057	0.365	0.486	Ns
Alpha	Avg Threshold	0.331 ± 0.047	0.320 ± 0.049	6153.5	-0.141	0.026	0.069	Ns
	Avg Edges	212.31 ± 18.07	214.26 ± 18.58	7656.5	0.03	0.635	0.726	ns
Beta	Avg Threshold	0.141 ± 0.022	0.152 ± 0.039	8724.5	0.152	0.017	0.066	ns
	Avg Edges	212.02 ± 15.25	212.94 ± 17.19	7238.5	-0.017	0.783	0.783	ns

Data are presented as Mean ± SD. HC, Healthy Controls (n = 154); ADS, Adolescents with depressive symptoms (n = 96). Avg Threshold indicates the mean adaptive phase lag index (PLI) threshold applied to individual functional connectivity matrices. Avg Edges represents the network density, defined as the absolute number of topological edges retained after thresholding*. U*, Mann-Whitney U statistic; *r*, effect size; *P*, uncorrected P-value; *P*_FDR_, P-value corrected using the False Discovery Rate method. *** indicates *P*_FDR_< 0.001; ns, not significant.

Based on these benchmark tests, this study confirms the biological validity of the constructed continuous brain network sequences in both the temporal evolution and spatial topology dimensions, thereby laying a solid and reliable data foundation for subsequent comprehensive analyses of the spatiotemporal dynamics and higher-order topological distortions of pathological networks across multiple scales (macro-, meso-, and micro-).

### 3.3 Macroscopic scale: whole-brain static connectivity, dynamic connectivity, and higher-order topological features

At the macroscopic, large-scale level (as shown in **Figures 3**–**5**), this study systematically evaluated the whole-brain network’s static functional connectivity (sFC), temporal dynamic evolution (dFC), and higher-order topological properties (TDA).

**Figure 3:**
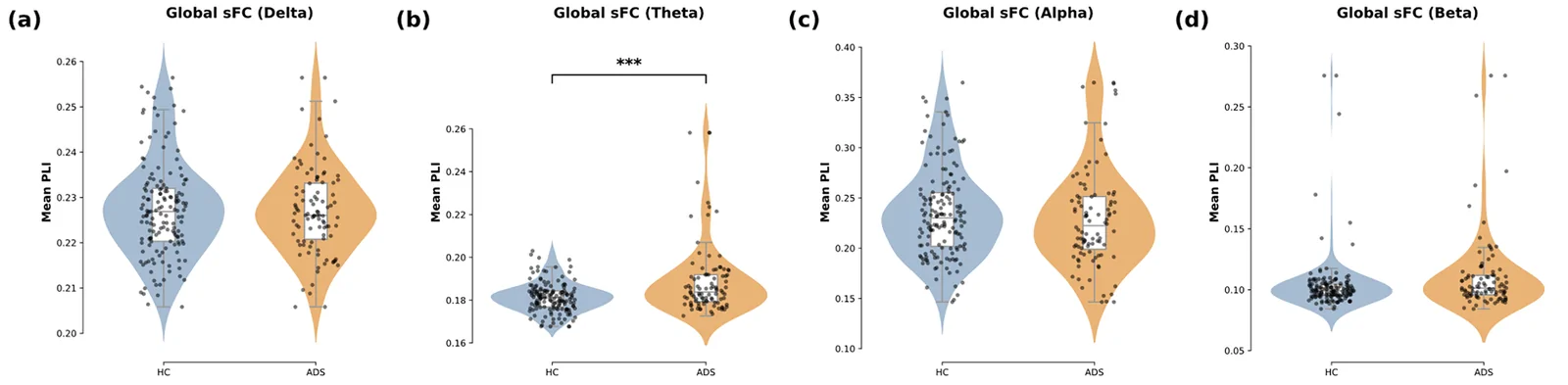
Comparison of intergroup distributions of whole-brain macroscopic static functional connectivity (sFC). Panels **(a–d)** show the distributions of the whole-brain average phase lag index (PLI) in the Delta, Theta, Alpha, and Beta frequency bands for the healthy control group (HC, blue) and the group of adolescents with depressive symptoms (ADS, orange), respectively. The figures use violin plots combined with underlying scatter plots to objectively present the distribution density, median, and overall dispersion of the data across groups. Asterisks indicate the significance levels of between-group statistical differences after correction for multiple comparisons (***PFDR< 0.001).

**Figure 4:**
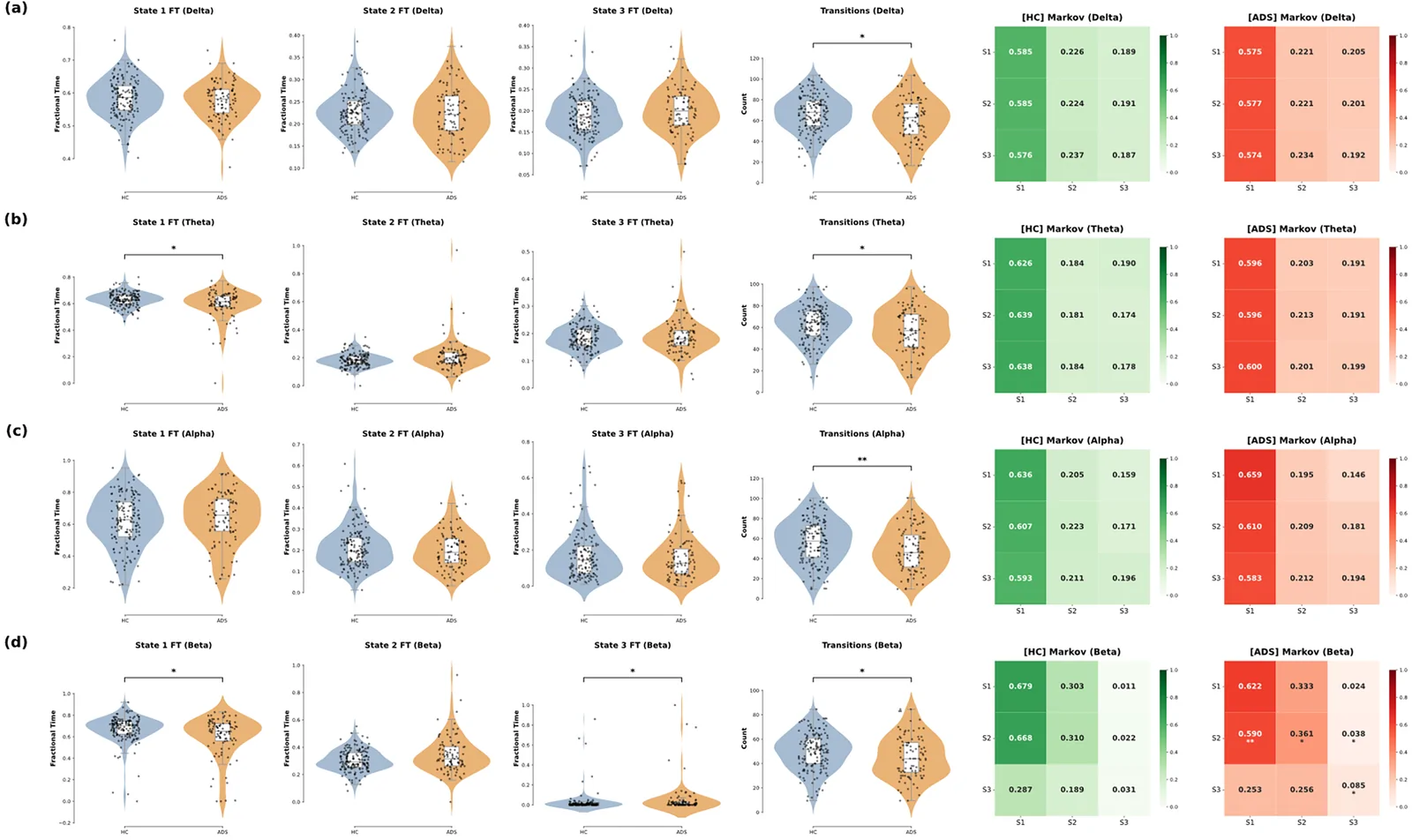
Parameters and Markov transition probability matrices of whole-brain dynamic functional connectivity states (dFCS). Panels **(a–d)** present dFCS characteristics during the dynamic evolution of the whole-brain network across four frequency bands. The left-hand side of each panel uses violin plots and scatter plots to display the fractional time and total transition frequency for the HC and ADS groups across three network dFCS (States 1–3); The heatmaps on the right side of each panel display the group-level Markov dFCS transition probability matrices (Markov Matrices), with values reflecting the probability of dFCS self-maintenance (diagonal) and the probability of cross-state transitions (off-diagonal). Asterisks indicate the significance levels of statistical differences between groups after correction for multiple comparisons (*PFDR< 0.05, **PFDR< 0.01).

**Figure 5:**
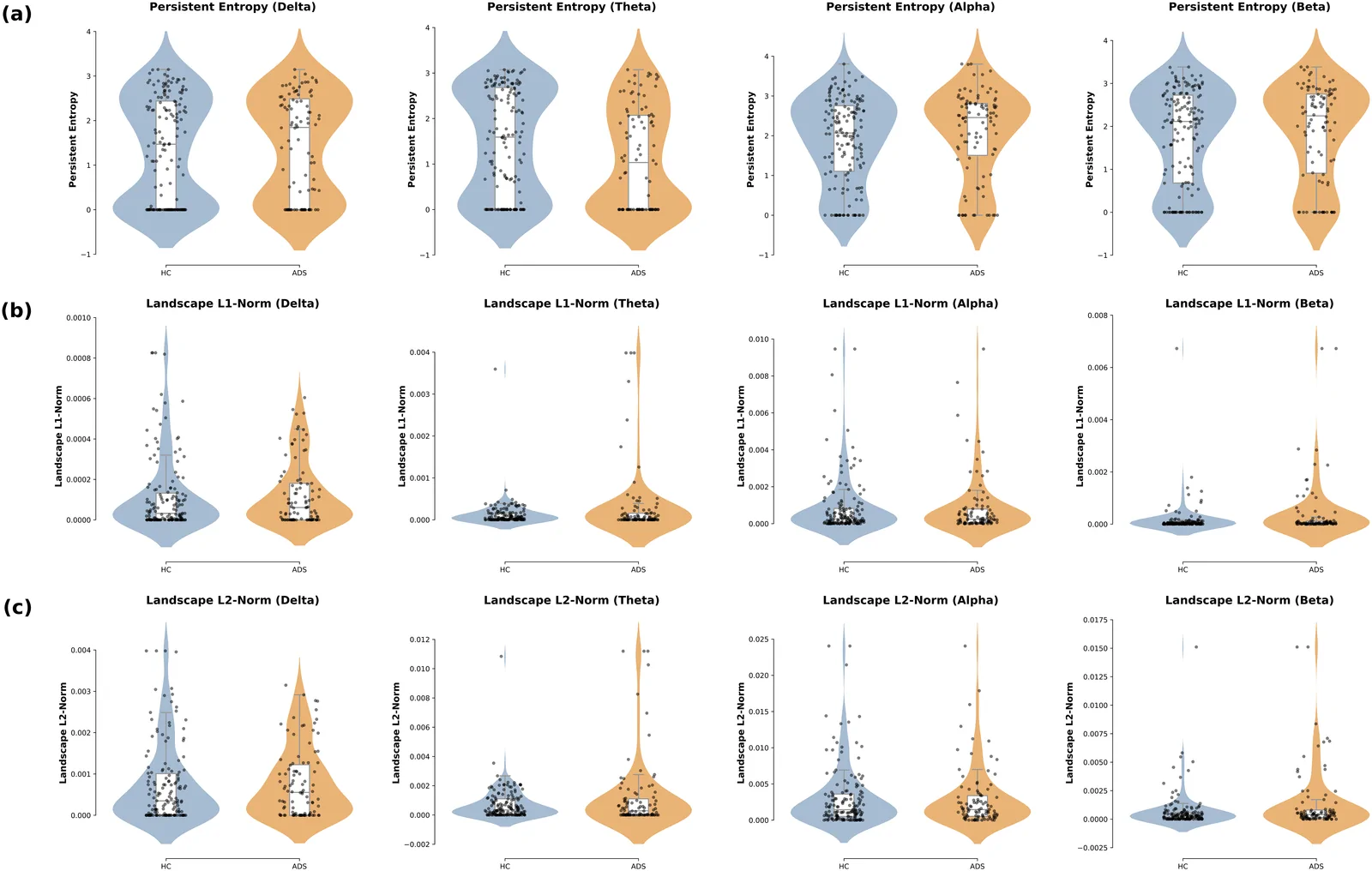
Distribution of higher-order topological features of the whole-brain macroscopic network based on topological data analysis (TDA). Panels **(a–c)** show the distribution of higher-order topological invariants extracted using the persistent homology algorithm across four frequency bands in the HC group (blue) and the ADS group (orange), respectively. The topological metrics shown include Persistent Entropy, which characterizes global one-dimensional synchrony group features, as well as the Landscape L1-Norm and Landscape L2-Norm of the first-level persistent landscape function. The specific numerical distributions of each metric and their outlier patterns are visualized using violin plots and underlying scatter plots.

Regarding static functional connectivity (sFC) (**Figure 3**; see **Supplementary Table S1** in the **Supplementary Materials**), global quantification results showed that baseline topological connectivity in the Delta and Alpha bands remained relatively constant across both groups; however, the ADS group showed a significant increase in global theta-band connectivity (HC: 0.181 ± 0.007, ADS: 0.189 ± 0.017, *P*_FDR_< 0.001). The distribution characteristics of the data indicate that the average phase lag index (PLI) in the Theta band for the healthy control group (HC) closely converged within the low range of 0.17 to 0.19, whereas the ADS group exhibited an overall rightward shift in connectivity load, with a significant upward shift in the median value and a large number of outlier values exceeding 0.22 and even reaching as high as 0.26. It is worth noting that although the ADS group exhibited a marginal increase in sFC in the beta band (uncorrected P = 0.031), this difference did not reach statistical significance after applying stringent cross-band multiple comparison correction (PFDR = 0.063).Crucially, the reference-independent sensitivity analysis utilizing fixed proportional thresholds (15%, 20%, and 25%) consistently replicated the significant over-coupling in the Theta band for the ADS group (all *P*_FDR_< 0.05; see **Supplementary Table S11** in the **Supplementary Materials**).

Prior to analyzing the temporal dynamics, a quantitative clustering validation was performed to determine the optimal number of dynamic functional connectivity states (dFCS). As shown in **Supplementary Figure S1**, the evaluation of $k=2$ to 8 revealed that while the Silhouette Score peaked at k=2, k=3 consistently maintained a robust secondary high plateau of clustering performance across all frequency bands, alongside a continuous decrease in the Sum of Squared Errors (SSE) curves. While purely distance-based mathematical metrics often favor an oversimplified binary partition (k=2), adopting a 2-state model in resting-state network analysis is neurobiologically inadequate. As emphasized by Preti et al. (6), dynamic brain networks operate across multiple stable functional configurations that reflect continuous cognitive and emotional adjustments. A binary model can only capture a crude dichotomy (e.g., high vs. low integration), failing to resolve the transient multi-stable transition bottlenecks characteristic of clinical psychopathology. In contrast, the 3-state dFCS (k = 3) model provided a parsimonious solution with sufficient resolution to characterize reduced transition flexibility and state-transition rigidity associated with depressive symptoms, while avoiding an overly simplified binary partition. Therefore, the 3-state model was established as the optimal parsimonious solution, achieving a rigorous balance between mathematical validity and neurobiological interpretability.

Building upon this established 3-state model, our whole-brain dynamic functional connectivity (dFC) analysis further quantified the characteristics of state transitions during network evolution (**Figure 4**; see **Supplementary Table S2** in the **Supplementary Materials**). The Mann–Whitney U test results showed that the ADS group had a significantly lower total number of state transitions than the HC group across all frequency bands (Delta, Theta, and Beta bands: *P*_FDR_< 0.05; Alpha band: *P*_FDR_< 0.01). The Mann–Whitney U test results further showed that fractional time (FT) exhibited significant band-specific shifts: compared with the HC group, the ADS group showed a significant shortening of the dwell time in State 1 (Theta band: HC 0.632 ± 0.053, ADS 0.597 ± 0.108, *P*_FDR_ = 0.035; Beta band: HC 0.671 ± 0.127, ADS 0.601 ± 0.189, *P*_FDR_ = 0.012), while the dwell time in State 3 in the Beta band was significantly prolonged (HC 0.026 ± 0.103, ADS 0.056 ± 0.159, *P*_FDR_ = 0.013).

The Markov transition probability matrix further indicated altered beta-band state-transition patterns in the ADS group. Specifically, the ADS group showed higher self-transition probabilities for States 2 and 3 (S2→S2: HC 0.310 vs. ADS 0.361, PFDR = 0.022; S3→S3: HC 0.031 vs. ADS 0.085, PFDR = 0.043). In addition, the transition probability from State 2 to State 1 was lower in the ADS group (S2→S1: HC 0.668 vs. ADS 0.590, PFDR = 0.007), whereas the transition probability from State 2 to State 3 was higher (S2→S3: HC 0.022 vs. ADS 0.038, PFDR = 0.020).

Regarding higher-order topological attributes (TDA) (**Figure 5**; see **Supplementary Table S3** in the **Supplementary Materials**), persistent entropy—which characterizes global geometric complexity—and the L1 and L2 norms of the landscape function were extracted based on persistent homology. After applying strict cross-band FDR correction, the results showed that the two groups did not exhibit statistically significant differences in global topological measures across all frequency bands (PFDR > 0.05). For example, differences in persistent entropy in the Theta band (PFDR = 0.057) and landscape norms in the Beta band were both statistically filtered as negative results.

To address the potential risk of false positives, a 100,000-iteration permutation and bootstrapping validation was performed on all initially significant macroscopic metrics. The validation results (see **Supplementary Table S14**) demonstrated that all 12 significantly altered macroscopic features (including Theta-band global sFC, total transitions, and Beta-band Markov transition probabilities) successfully survived the stringent family-wise non-parametric penalty (Permutation *P*_FDR_< 0.05), with their Bootstrap 95% confidence intervals entirely excluding zero.

In summary, objective data indicate that macroscopic abnormalities in the ADS brain are specifically anchored to static over-coupling in the Theta band and widespread dynamic transition rigidity across frequency bands; meanwhile, global higher-order topological structures maintain apparent constancy when averaged across macroscopic space.

### 3.4 Mesoscale: static and dynamic connectivity and local topological features at the brain region level

After identifying global network abnormalities at the macroscopic scale, this section further localizes the specific anatomical origins of the aforementioned connections and topological features at the mesoscale.

Regarding static functional connectivity (sFC) (**Figure 6**; see **Supplementary Table S4** in the **Supplementary Materials**), the intergroup difference matrix based on 5×5 anatomical regions (frontal, central, temporal, parietal, and occipital lobes), the inter-group difference matrix revealed that, following strict FDR correction via 60 independent cross-region and cross-frequency band tests, mesoscale network abnormalities in the adolescents with depressive symptoms (ADS) group exhibited high frequency-band specificity, with significant abnormal hyperconnectivity largely concentrated in the theta band. Specifically, in the Theta band, the ADS group exhibited an extensive hypersynchronized network centered on the central region (Central): not only was connectivity within the central region significantly elevated (r = 0.296, PFDR = 0.002), but it also displayed extremely strong inter-regional interactions, including central region–frontal lobe (r = 0.314, PFDR = 0.002), Central-parietal (r = 0.281, PFDR = 0.004), and Central-temporal (r = 0.235, PFDR = 0.018) regions. Furthermore, this high connectivity in the theta band also extended to within the frontal lobe (r = 0.253, PFDR = 0.012), within the parietal lobe (r = 0.206, PFDR = 0.044), and between the parietal and temporal lobes (r = 0.230, PFDR = 0.019). In the alpha band, the ADS group exhibited focal hypoconnectivity in the visual network, including significant attenuation within the occipital lobe (r = -0.235, PFDR = 0.018) and in the occipital-parietal interaction (r = -0.204, PFDR = 0.044). In the beta band, only a significant increase in local temporal-central connectivity was observed (r = 0.200, PFDR = 0.046).

**Figure 6:**
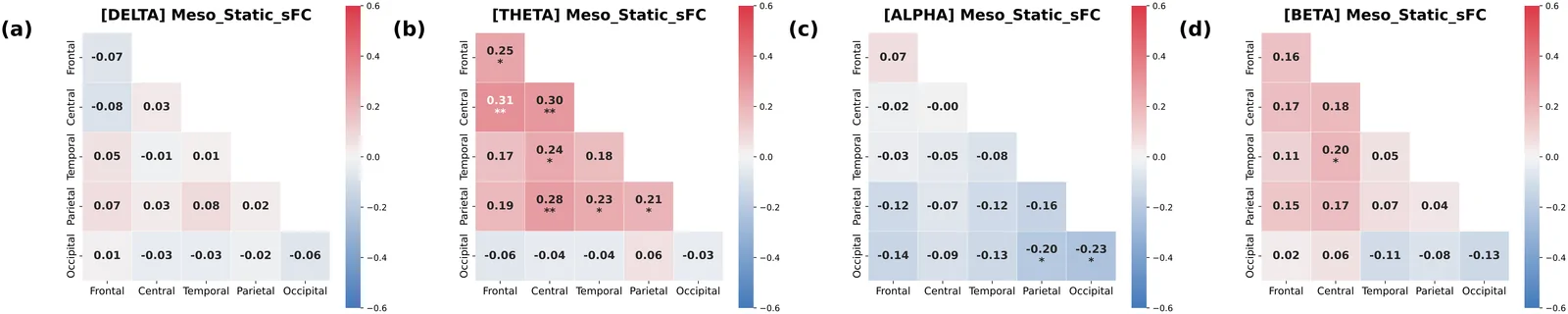
Matrix of between-group differences in static functional connectivity (sFC) at the mesoscale. Panels **(a–d)** present the effect size r matrices for between-group sFC differences (ADS vs. HC) based on 5×5 anatomical brain regions (frontal, central, temporal, parietal, and occipital) in the Delta, Theta, Alpha, and Beta frequency bands, respectively. The lower triangular part of the matrix shows the differences between intra-regional connections (diagonal elements) and inter-regional connections; the color mapping reflects the magnitude and direction of the effect size r (positive values/red indicate stronger connections in the ADS group, while negative values/blue indicate stronger connections in the HC group). Asterisks indicate connections that are statistically significant after FDR correction (*PFDR< 0.05, **PFDR< 0.01).

With regard to whole-brain temporal dynamics of connectivity (dFC) (**Figure 7**; see **Supplementary Table S5** in the **Supplementary Materials** for details), the regional coefficient of variation (CV) matrix quantifies the temporal fluctuations in connection strength between regions. Unlike the widespread network abnormalities observed in sFC, between-group differences in dFC exhibit extreme frequency-specific and spatial focal patterns. After global multiple comparisons with FDR correction, the only feature retaining statistical significance was located in the delta band: the ADS group exhibited a significantly higher coefficient of variation in temporal variability for frontal-frontal connections than the healthy control (HC) group (r = 0.278, PFDR = 0.013). To verify whether this mesoscale dynamic instability was driven by differences in baseline connectivity, a supplementary validation utilizing the absolute temporal standard deviation (Absolute SD) was conducted. Consistent with the primary CV findings, the absolute temporal fluctuation within the frontal-frontal network in the delta band remained significantly elevated in the ADS group (*P*_FDR_= 0.002; see **Supplementary Table S12**), indicating that this difference is independent of baseline mean connectivity. In the Theta, Alpha, and Beta frequency bands, no statistically significant differences were observed in the dynamic fluctuation indices within the remaining mesoscale regions or across regions (PFDR > 0.05 for all edges).

**Figure 7:**
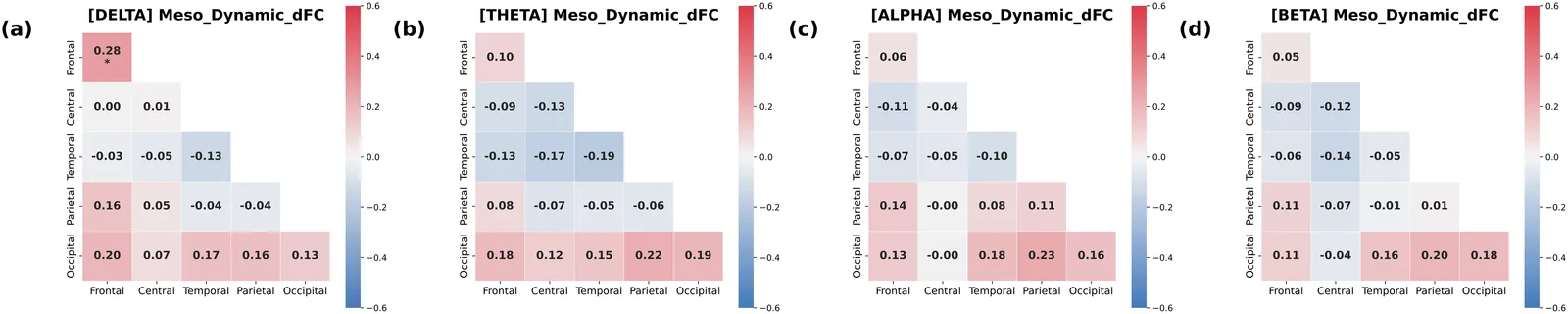
Matrix of between-group differences in dynamic functional connectivity (dFC) at the mesoscale. Panels **(a–d)** show the effect size r matrices for between-group differences in dynamic temporal variability coefficients (ADS vs. HC) based on 5×5 anatomical brain regions in the Delta, Theta, Alpha, and Beta frequency bands, respectively. The matrices illustrate the degree of difference between intra-regional (diagonal) and inter-regional (off-diagonal) connections; the color mapping reflects the magnitude and direction of the effect size r (positive values/red indicate greater dynamic temporal variability in the ADS group, while negative values/blue indicate less variability). Asterisks indicate statistical significance after FDR correction (*P_FDR_< 0.05).

Regarding mesoscale higher-order topological features (TDA) (**Figure 8**; see **Supplementary Table S6** in the **Supplementary Materials** for details), this study extracted the region-averaged normalized node persistence (Meso_NP) across four frequency bands for five anatomical brain regions. After joint correction for a total of 20 multiple comparisons across frequency bands and brain regions, the results showed that the two groups did not exhibit statistically significant differences in the average NP metrics across all frequency bands and all anatomical brain regions (PFDR > 0.05 for all regions). The intergroup medians and overall data distribution patterns for each anatomical region showed high overlap on the violin plot.

**Figure 8:**
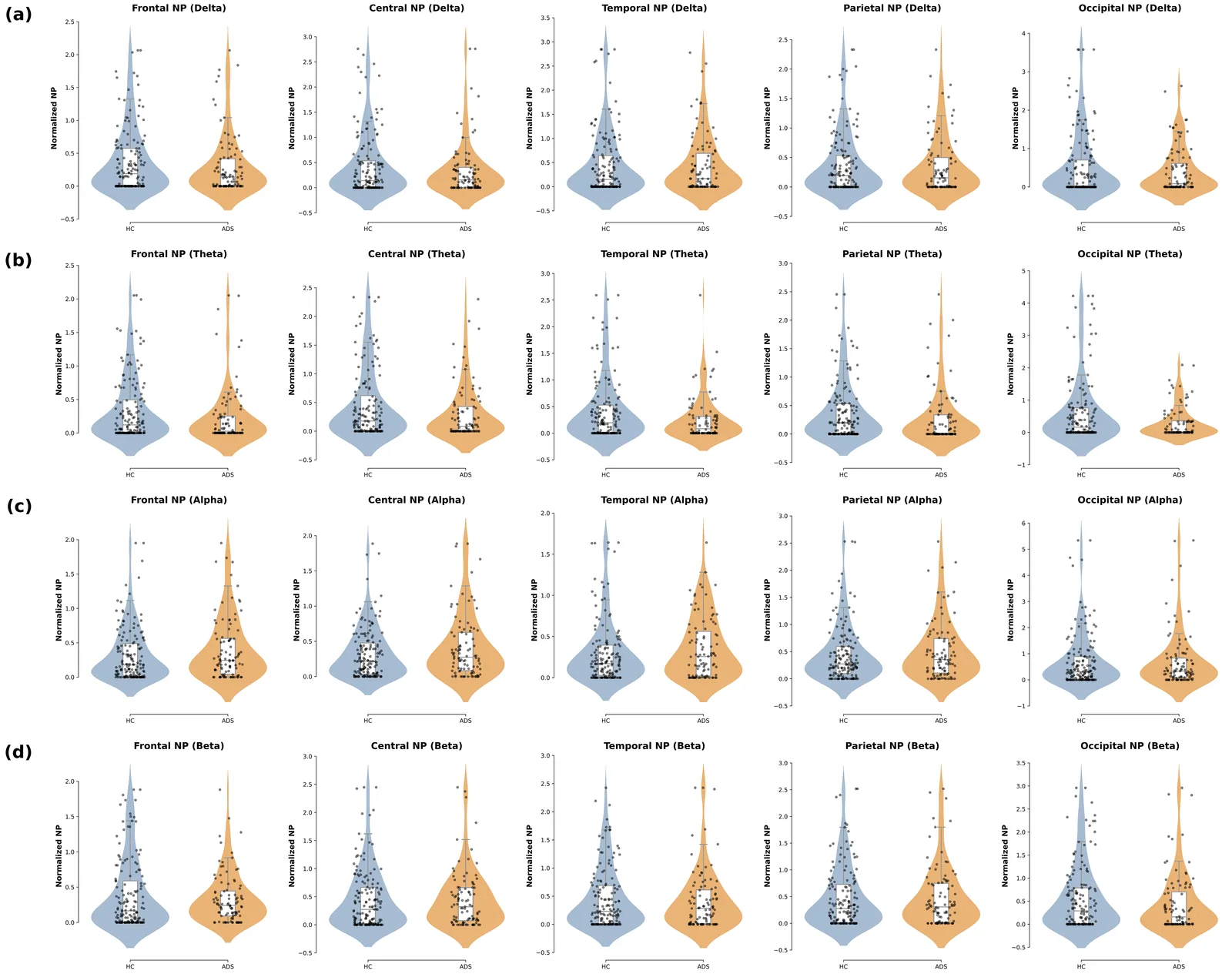
Intergroup differences in node persistence (NP) at the mesoscale. Panels **(a–d)** present the distribution of normalized node persistence (NP) metrics across four frequency bands in five anatomical brain regions, respectively. The figures use violin plots combined with underlying scatter plots to objectively compare the density, median, and interquartile range of NP metrics in each brain region between the HC group (blue) and the ADS group (orange).

Consistent with the validation at the macroscale, the 100,000-iteration permutation and bootstrapping results for the mesoscale network (see **Supplementary Table S15**) confirmed the statistical stability of the aforementioned regional alterations. Under the 60-dimensional family-wise penalty, all 11 initially significant mesoscale functional connections maintained strict statistical significance (all Permutation *P*_FDR_< 0.05), with their Bootstrap 95% confidence intervals entirely excluding zero.

In summary, based on these objective data, after excluding false-positive interference caused by multiple comparisons across spatial and frequency domains, the mesoscale pathological features of the ADS brain are precisely anchored to widespread static over-coupling in the Theta band—with the central region serving as the network hub—as well as abnormally intensified phasic dynamic fluctuations in the Delta band prefrontal network; Meanwhile, the higher-order geometric topological features (NP) of each anatomical region maintained consistency in intergroup representations; this masking effect objectively prompted this study to delve further into the microscopic scale with higher spatial resolution.

### 3.5 Microscale: edge-level static and dynamic connectivity and node-level topological characteristics

To overcome the spatial averaging effects of mesoscale anatomical regions, this section further conducted a comprehensive *in situ* decomposition of static coupling strength, temporal coefficient of variation, and higher-order geometric-topological features at the microscopic sensor scale (a whole-brain 30 × 30 electrode node matrix), and performed rigorous joint correction for cross-frequency band and full-space multiple comparisons (global FDR).

Intergroup differences in static functional connectivity at the microscopic scale (as shown in **Figure 9**; see **Supplementary Table S7** for significant edges and **Supplementary Dataset 1** for full statistical data) exhibited an extremely focal spatial distribution following global correction via 1,740 independent edge tests. Significant hyperconnectivity in the adolescents with depressive symptoms (ADS) group was specifically confined to the theta band. Specifically, only two connections across the entire brain exhibited a statistically significant increase in coupling strength, both precisely anchored within the midline network connecting the medial prefrontal cortex to the central motor cortex: the left frontal pole to the midline central region (Fp1–FCz: r = 0.304, PFDR = 0.046) and the midline prefrontal cortex to the midline central region (Fz–FCz: r = 0.316, PFDR = 0.046). In the Delta, Alpha, and Beta bands, none of the whole-brain micro-sFC edges showed FDR-corrected significant differences (PFDR > 0.05).

**Figure 9:**
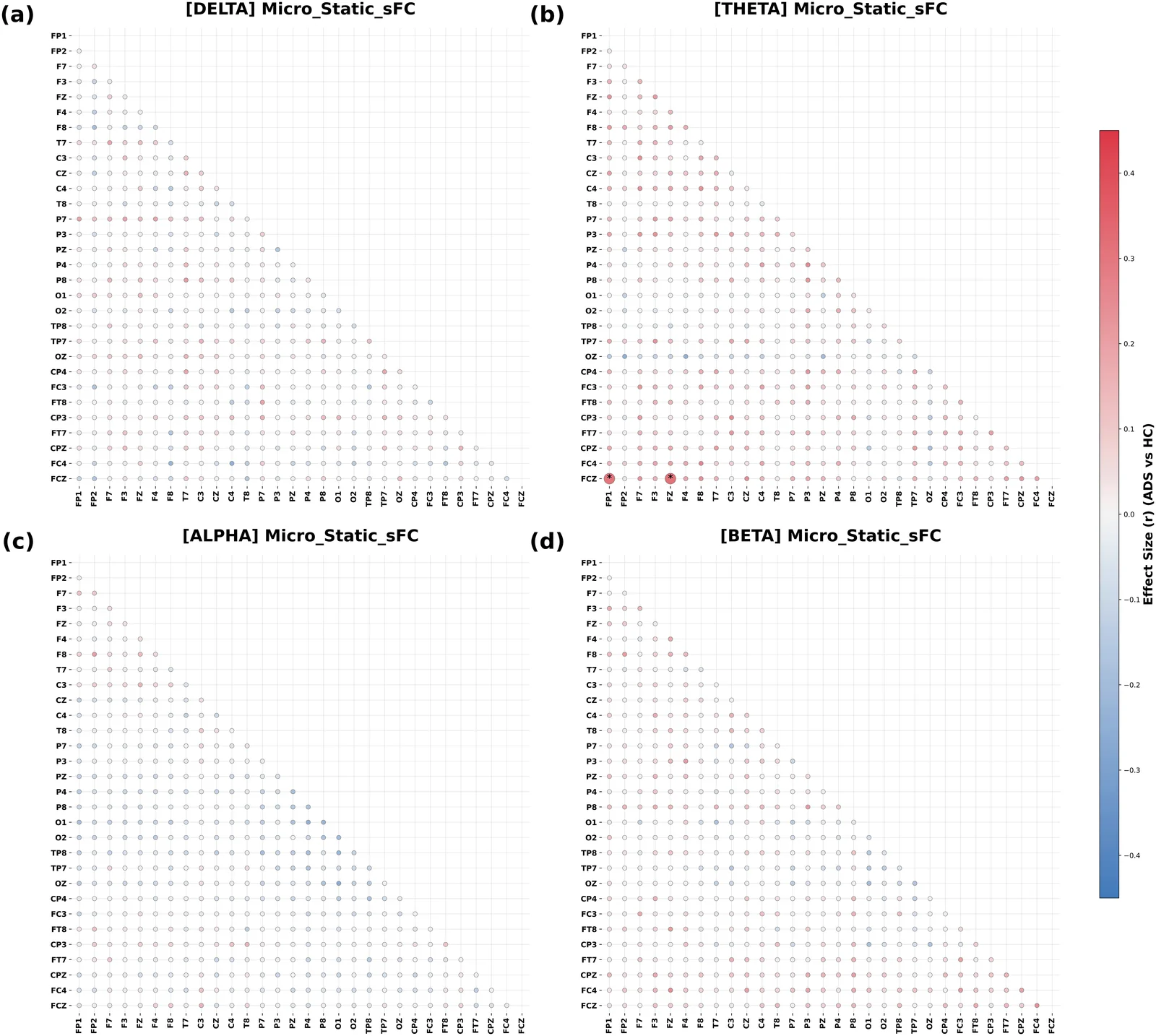
Bubble plots of intergroup differences in static functional connectivity (sFC) at the micro-scale. Panels **(a–d)** show triangular bubble plots of the effect size $r$ for intergroup differences (ADS vs. HC) in sFC between 30×30 whole-brain electrode nodes in the Delta, Theta, Alpha, and Beta frequency bands, respectively. The shade and orientation of the bubbles correspond to the magnitude of the effect size r (red indicates abnormally increased connectivity in the ADS group, r > 0; blue indicates abnormally decreased connectivity, r< 0); the size of the bubbles strictly corresponds to the level of statistical significance after multiple comparison correction (the smaller the PFDR value, the larger the bubble). Asterisks indicate connections that are statistically significant after FDR correction ( *P_FDR_< 0.05).

The coefficient of variation in the temporal variability of microscale dynamic functional connectivity (as shown in **Figure 10**; see **Supplementary Table S8** for significant connections and **Supplementary Dataset 2** for complete statistical data) also exhibited highly convergent localized abnormalities after global correction. In the Theta band, the ADS group exhibited specific long-range dynamic interaction instability, with significantly different connections precisely originating in the right prefrontal cortex and establishing a longitudinal projection pathway to the midline visual cortex in the occipital lobe: including the right dorsolateral prefrontal cortex to the midline occipital lobe (F4 - Oz: r = 0.323, PFDR = 0.031) and from the right frontal pole to the midline occipital cortex (Fp2–Oz: r = 0.308, PFDR = 0.036). Consistent with the mesoscale validation approach, given the elevated mean static connectivity (sFC) in the theta band among the ADS group, absolute temporal standard deviation (Absolute SD) was evaluated to rule out mean-driven arithmetic artifacts in the CV metric. The supplementary validation demonstrated that the absolute temporal oscillations of these specific prefrontal-occipital pathways (F4-Oz and Fp2-Oz) remained significantly higher in the ADS group (both *P*_FDR_< 0.01; see **Supplementary Table S12**). In all other frequency bands and across the vast majority of microscopic connections throughout the brain, differences in dynamic fluctuations were filtered out as negative results (PFDR > 0.05).

**Figure 10:**
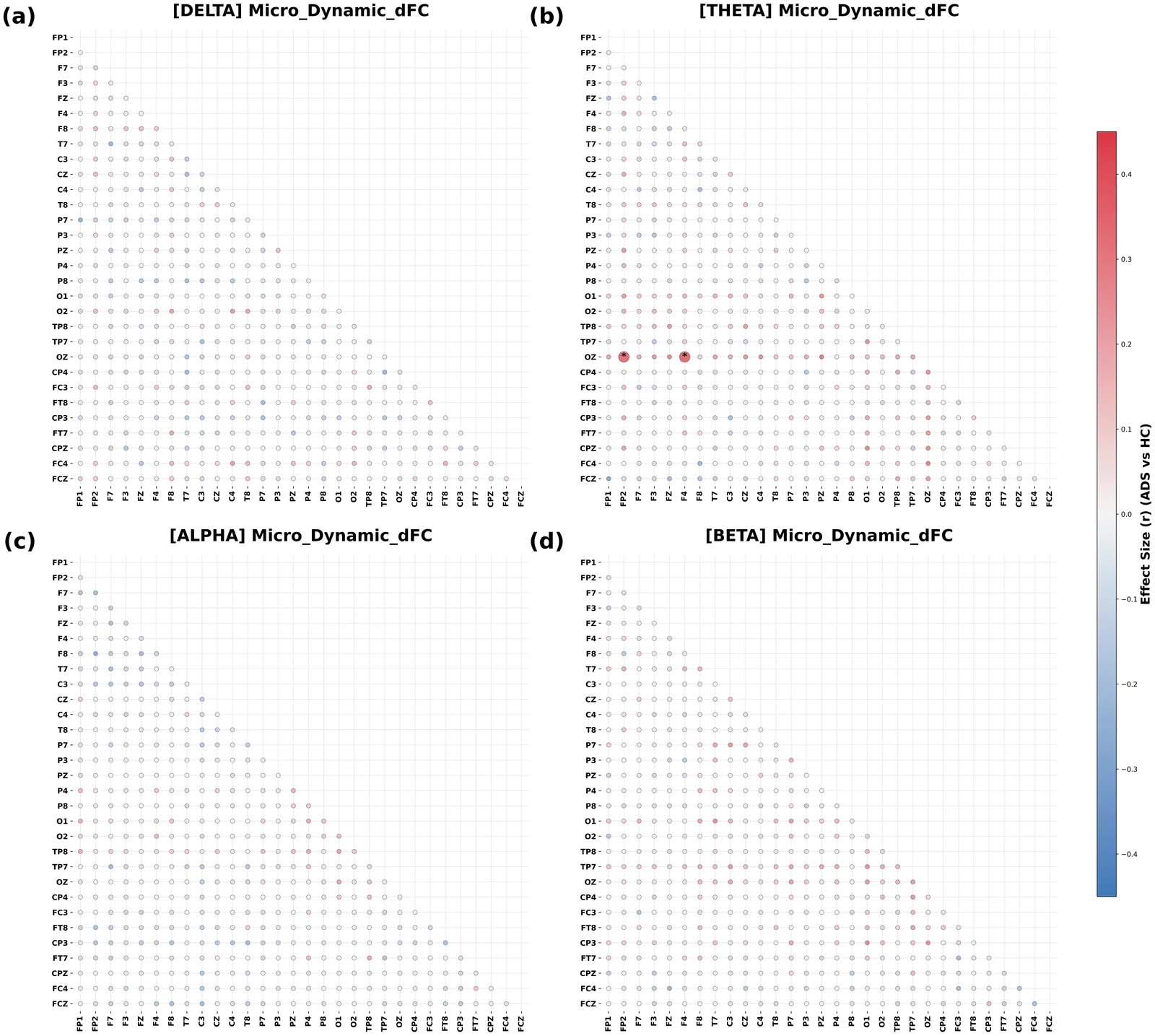
Bubble plot of between-group differences in temporal variability of dynamic functional connectivity at the micro-scale, at the electrode-edge level. Panels **(a–d)** show triangular bubble plots of the effect size $r$ for between-group differences (ADS vs. HC) in the coefficient of temporal variability of dynamic functional connectivity among whole-brain electrodes in the Delta, Theta, Alpha, and Beta frequency bands, respectively. Bubble color reflects the magnitude of the effect size $r$ (red bubbles indicate that the ADS group exhibits higher dynamic temporal variability compared to the HC group, $r > 0$; blue indicates reduced variability, $r< 0$); bubble size strictly corresponds to the level of statistical significance (the smaller the PFDR, the larger the bubble). Asterisks denote connections that are statistically significant after FDR correction ( *P_FDR_< 0.05).

Higher-order features of microscopic nodes (NF and NP) extracted based on sustained synchrony (as shown in **Figure 11**; see **Supplementary Table S9** for significant nodes and **Supplementary Dataset 3** for complete statistical data) revealed highly localized geometric distortions after global FDR correction following 480 feature tests. In the theta band, topological closed-loop features in the ADS group exhibited significant attenuation and collapse, with the abnormal focus precisely localized to the right prefrontal region: the right frontal pole (Fp2: NF r = -0.202, *P*_FDR_ = 0.048; NP also showed a significant decrease) and the right dorsolateral prefrontal cortex (F4: NF r = -0.203, *P*_FDR_ = 0.048; NP r = -0.227, *P*_FDR_ = 0.032), where higher-order topological properties were significantly suppressed. In contrast, in the alpha band, the ADS group exhibited significant overactivation of topological features in the left lateral temporal lobe, with both frequency and persistence at the FT7 node showing significant increases (FT7: NF r = 0.254, *P*_FDR_ = 0.032; NP r = 0.223, *P*_FDR_ = 0.048).

**Figure 11:**
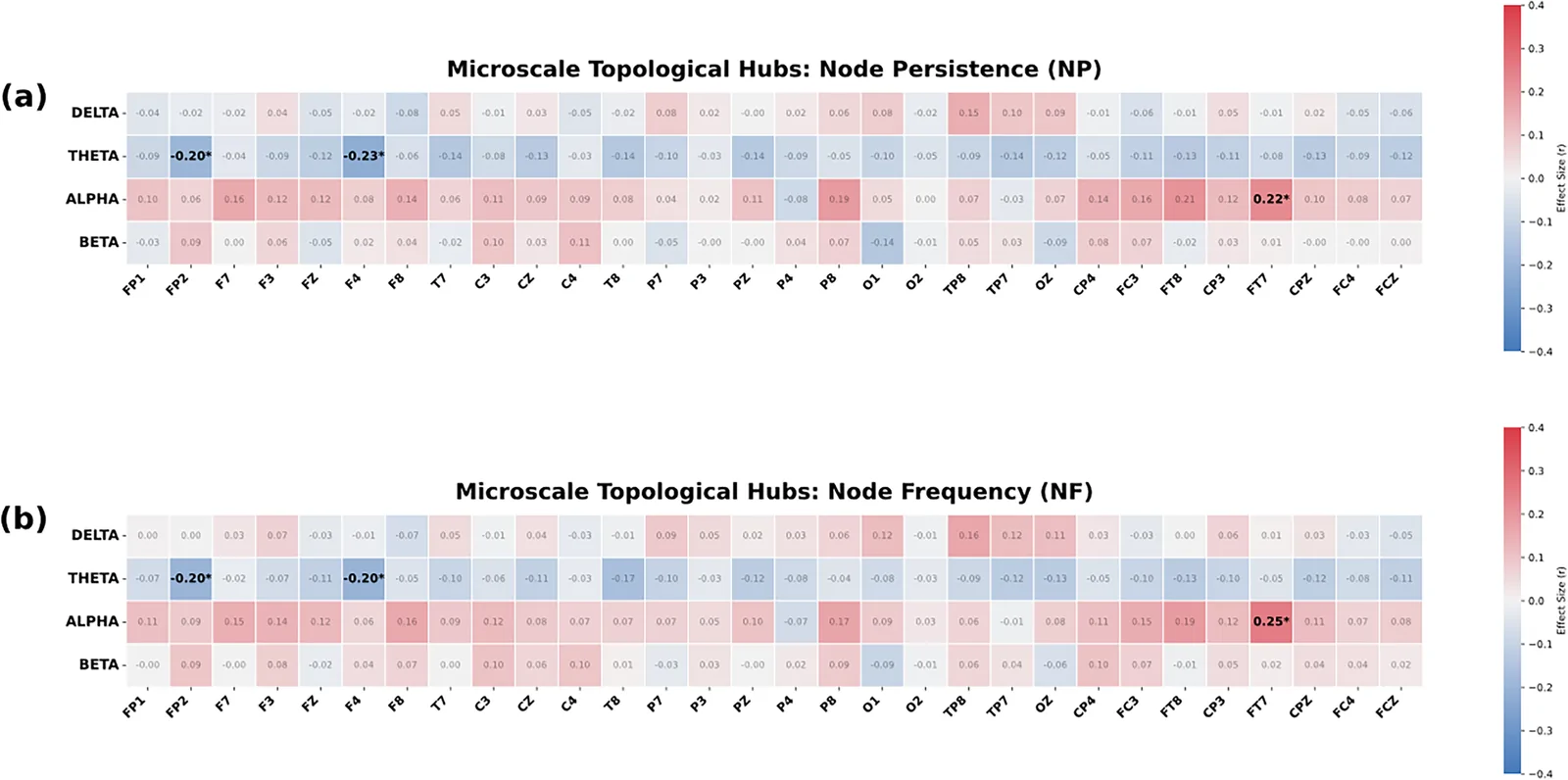
Band-specific spatial distribution of node topological features (NP and NF) at the microscale. Panels **(a, b)** show the effect size r maps of node persistence (NP) and topological frequency (NF) between groups (ADS vs. HC) for 30 whole-brain electrode nodes across four frequency bands, respectively. The figures quantify the higher-order topological closed-loop features of each electrode node, with red indicating a positive shift (r > 0) and blue indicating a negative shift (r< 0). Black asterisks denote connections that are statistically significant after FDR correction ( *P_FDR_< 0.05).

The subsequent 100,000-iteration permutation and bootstrapping validation (see **Supplementary Table S16**) demonstrated that 18 out of the 19 initially significant microscale features maintained statistical significance (Permutation *P*_FDR_< 0.05$, Bootstrap 95% CIs excluding zero). Notably, under the family-wise penalty (N = 1,740), the static edge between Fp1 and FCz shifted to a trend-level effect (Permutation *P*_FDR_ = 0.078).

In summary, the microscale analysis revealed spatially localized network alterations that were not fully captured at the macro- or meso-scale levels. Theta-band static over-coupling was mainly observed in the prefrontal–central connection, particularly Fz–FCz, whereas increased dynamic connectivity variability was mainly observed along right prefrontal–occipital pathways, including Fp2/F4–Oz. Higher-order topological alterations were further observed in right prefrontal nodes, particularly Fp2 and F4, while the left temporal node FT7 showed alpha-band topological alterations.

### 3.6 Spatial mapping of multidimensional abnormal features and exploratory scalp-level network hubs

In this study, we extracted the significance of statistical tests for 30 whole-brain electrode nodes across four feature indices (node static connectivity sFC, node temporal variability dFC, node persistence NP, and node frequency NF) and constructed a whole-brain multidimensional pathological evidence convergence matrix (as shown in **Figure 12**; see **Supplementary Table S10** for a list of significant features, and **Supplementary Dataset 3** for complete feature-level test data).The results showed that in the Delta and Beta frequency bands, none of the 30 whole-brain nodes exhibited significant feature-level alterations meeting the FDR multiple comparison correction (*P*_FDR_< 0.05) across the four feature indices. Intergroup abnormalities were primarily concentrated in the Theta band; in the Alpha band, the left temporofrontal node FT7 showed significant differences (*P*_FDR_< 0.05) in the two related topological indices, NP and NF.

**Figure 12:**
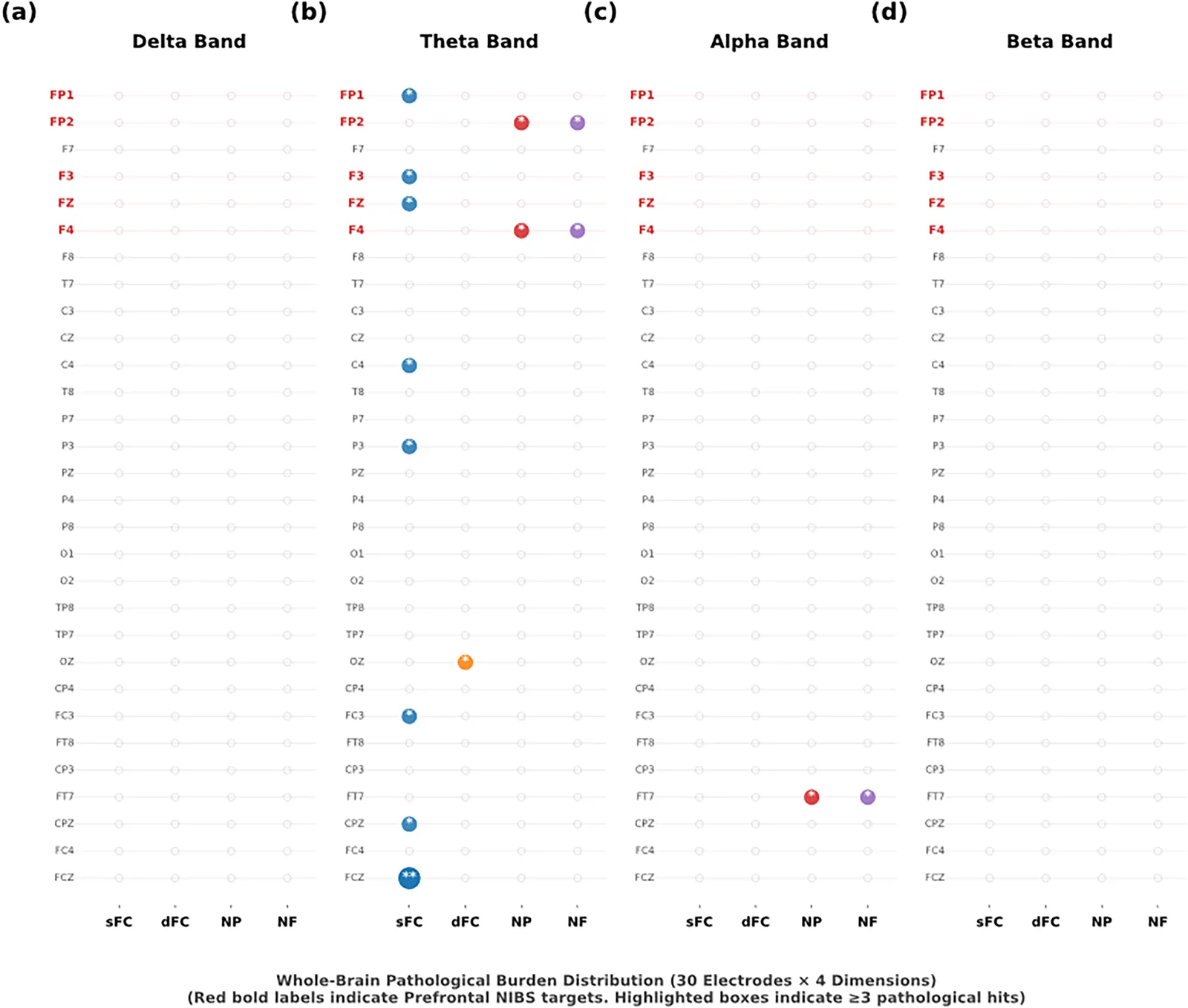
Matrix of convergent multidimensional pathological evidence across all brain electrodes. This matrix summarizes the node-level distribution of abnormal network features across 30 scalp electrodes and four frequency bands. Panels **(a–d)** correspond to the delta, theta, alpha, and beta bands, respectively. In each panel, rows represent scalp electrodes, and columns represent four network feature indices: nodal static connectivity strength (sFC), nodal dynamic connectivity variability (dFC), node persistence (NP), and node frequency (NF). A filled colored dot indicates a feature-level “hit,” defined as an ADS–HC group difference reaching FDR-corrected significance for that electrode, frequency band, and feature index (*P*_FDR_< 0.05). Empty gray circles indicate no FDR-corrected significant difference. Dot colors correspond to the feature index: blue = sFC, orange = dFC, red = NP, and purple = NF. Asterisks denote corrected significance levels (*P_FDR_< 0.05, **P_FDR_< 0.01). Bold red electrode labels indicate the five exploratory prefrontal scalp electrodes (Fp1, Fp2, F3, Fz, and F4). Multiple filled dots within the same electrode row indicate multi-feature convergence at the scalp-electrode level, rather than independent pathological dimensions or validated neuromodulation targets. NP and NF are related topological indices derived from the same H_1_ persistent-homology computation and should not be interpreted as fully independent dimensions.

Within the core Theta band, the spatial distribution of hits across the four dimensions and their levels of statistical significance exhibited the following characteristics: Notably, in contrast to the extremely sparse between-group differences at the micro-edge level (Edge-level sFC), a total of 8 significantly anomalous nodes were observed on the static connectivity (sFC) dimension, which reflects global integrative efficiency of nodes. Among these, FCz reached a high level of significance (*P*_FDR_< 0.01), while the remaining 7 nodes (Fp1, F3, Fz, C4, P3, FC3, CPz) reached the significance level (*P*_FDR_< 0.05). In the node temporal variability (dFC) dimension, only the occipital region node Oz recorded a significant hit (*P*_FDR_< 0.05); For the topological indices, NP and NF showed spatially overlapping significant alterations at Fp2 and F4 in the theta band (*P*_FDR_< 0.05). Because NF and NP were both derived from the same H_1_ persistent-homology computation, we further examined their dependence at these nodes. Spearman correlation analysis showed that NF and NP were highly correlated at Fp2 (*ρ* = 0.981, *P* < 0.001) and F4 (*ρ* = 0.980, *P* < 0.001; **Supplementary Table S18**). These results indicate strong statistical dependence between NF and NP at the two nodes showing concurrent topological alterations.

For the five prefrontal scalp electrodes highlighted in red in the figure (Fp1, Fp2, F3, Fz, F4), the matrix mapping revealed that all exhibited sensor-level group differences in the theta band, but with distinct feature profiles. Fp1, F3, and Fz showed significant abnormalities only in the static functional connectivity (sFC) index (*P*_FDR_< 0.05). In contrast, Fp2 and F4 did not show significant sFC abnormalities, but both exhibited convergent alterations across two related persistent-homology-derived topological indices, namely node persistence (NP) and node frequency (NF). Therefore, the Fp2/F4 findings should be interpreted as a related topological feature profile rather than as evidence for two independent abnormal dimensions. Taken together, these data indicate that the five prefrontal scalp electrodes were all represented among theta-band abnormal nodes at the micro-network level, exhibiting a complementary pattern of feature-level separation.

Furthermore, it is worth noting that certain brain regions not preset as prefrontal *a priori* targets (not marked in red) showed distinct feature profiles in the convergence matrix: First, the central region node FCz was the only sFC abnormal node in the entire brain to reach a high level of significance (*P*_FDR_< 0.01); Second, the occipital hub Oz was the only node in the entire brain to exhibit a significant anomaly in temporal variability (dFC); Third, in the alpha band, the left temporofrontal node FT7—also a non-target node—showed concurrent significance in the two related topological indices NP and NF.

A bootstrap resampling analysis (1,000 iterations) of the static functional connectivity (sFC) based Most Significant Connections (MSCs) yielded an average Jaccard index overlap rate of 88.80% (range: 35.71% to 100.00%). For the specific nodes identified in the multidimensional convergence matrix, the node-level stability analysis showed the following system lock probabilities: FCz (97.10%), P3 (95.10%), Fz (95.00%), C4 (93.50%), FT7 (92.80%), FC3 (88.10%), Fp1 (83.60%), Oz (81.20%), and F3 (79.00%). The extraction probabilities for Fp2 and F4 were 0.00% and 24.60%, respectively (see **Supplementary Table S13** for complete stability data of all 30 electrodes).Furthermore, all significantly altered nodal features incorporated into the multidimensional convergence matrix successfully passed the 100,000-iteration permutation validation (Permutation *P*_FDR_< 0.05; see **Supplementary Table S16**).

## 4 Discussion

This study developed a cross-scale, multidimensional analytical framework integrating adaptive thresholding, static-dynamic evolution, and sustained synchrony, and systematically explored the patterns of network remodeling in adolescents with depressive symptoms (ADS) from the macroscopic global level to the microscopic *in situ* level. The core findings of this study preliminarily corroborate the initial hypothesis: First, brain network alterations in ADS exhibit a relatively pronounced “spatial scale dependence”; widespread compensatory effects at the macroscopic and mesoscopic levels may, to some extent, mask higher-order topological impairments in micro-focal core hubs; Second, the pathological convergence map constructed based on multidimensional features not only meticulously reveals the potential dimensional heterogeneity of damage within classic prefrontal regulatory targets but also provides clues for identifying potential regulatory centers that lie outside traditional *a priori* frameworks. In summary, this multidimensional analytical framework and the network patterns it reveals offer a new observational perspective for unraveling the complex pathological evolution of adolescent depression and, to some extent, provide theoretical insights for the subsequent optimization of clinical targets.

### 4.1 Adaptive thresholding and stability of EEG network construction

In brain network analysis, the choice of threshold directly influences the topological structure of functional networks and their subsequent statistical interpretation. How to minimize subjective bias in threshold selection while controlling for noise connections while preserving valid neural connections is a key methodological issue in resting-state brain network research. Previous studies have shown that resting-state functional connectivity is not a fixed, static structure, but rather exhibits distinct non-stationarity and transient spatiotemporal fluctuations (2, 5). Consequently, traditional absolute threshold or fixed-ratio threshold methods may struggle to adequately accommodate the high temporal resolution of EEG signals: during periods of low synchronization, fixed thresholds may result in an overly sparse network or even localized topological fragmentation; conversely, during periods of high synchronization, they may retain too many low-specificity or noise-related connections, thereby compromising the stability and physiological interpretability of network structure estimates (6, 13).

Based on these considerations, this study employs a data-driven adaptive thresholding strategy to construct subject-specific brain functional networks. Rather than presetting a uniform absolute threshold or fixed network density, this method dynamically determines thresholds based on the distribution characteristics of the connectivity matrix within each time window, thereby better adapting to the baseline fluctuations of resting-state EEG functional connectivity over time (1). The results show that although network density exhibits a certain degree of alternation between sparse and dense states across different time segments, the thresholded brain functional network still maintains a relatively stable core connectivity structure. This indicates that the adaptive thresholding strategy can filter out low-intensity nonspecific connections while preserving the network’s basic topological integrity, providing a relatively robust foundation for subsequent characterization of the dynamic reorganization features of brain networks in adolescents with depressive symptoms.

Furthermore, human functional brain networks typically exhibit small-world topological characteristics, meaning they maintain high local clustering while preserving short global information transmission paths. This structure is believed to help maintain a balance between local functional specialization and whole-brain information integration (14). Therefore, whether the thresholded network retains its small-world properties is one of the key criteria for evaluating the validity of the brain network construction. In this study, we further employed dual small-world normalization metrics—SW1 and SW2—to validate the topological validity of the thresholded functional brain networks. The results show that the functional brain networks constructed for all participants retained small-world properties, suggesting that the adaptive thresholding strategy did not transform the networks into random or regular structures lacking physiological significance, but rather preserved, to a certain extent, the inherent complex topological organization of brain networks (15).

Overall, the adaptive thresholding results and the validation of small-world properties jointly support the robustness and physiological plausibility of the brain functional network construction method used in this study (16). This method provides a reliable foundation for subsequent analyses of static and dynamic functional connectivity as well as higher-order topological features, and reduces the likelihood that any subsequently identified focal network abnormalities are attributable to threshold selection bias or network construction strategies. Based on this methodological premise, this study further examines the patterns of abnormalities in the brain functional networks of adolescents with depressive symptoms across macroscopic, mesoscopic, and microscopic scales.

### 4.2 Macroscopic brain network abnormalities: systemic connectivity compensation and dynamic rigidity

At the macro level, this study found that brain network abnormalities in adolescents with depressive symptoms (ADS) did not manifest as widespread disruption of global topological structure, but were primarily characterized by enhanced static functional connectivity in the theta band and reduced flexibility in dynamic state transitions. Specifically, the ADS group exhibited an elevated median value for static functional connectivity in the theta band, accompanied by a higher frequency of high connectivity strength values, suggesting that their resting-state brain network may exhibit characteristics of low-frequency excessive synchronization (17). Previous studies have shown that depressive disorders are closely associated with abnormalities in low-frequency brain oscillations, such as theta, and brain functional connectivity; Theta rhythms are also believed to be involved in internal attention, emotional processing, memory retrieval, and cognitive control processes (18). Although both subclinical ADS and severe clinical MDD populations exhibit prominent theta-band hyper-synchronization, their mechanistic implications are profoundly different. In severe MDD, excessive low-frequency coupling typically reflects the functional decompensation and irreversible stalling of cognitive control networks, often manifesting behaviorally as entrenched pathological rumination. In stark contrast, the unique phenotype of subclinical depression perfectly aligns with the “overcompensation” hypothesis in early-stage psychopathology (19), which posits that the subclinical brain sustains core functioning through hyper-connectivity before the network ultimately succumbs to the structural degradation typical of major depression. Therefore, the excessive coupling in the Theta band observed in this study may reflect enhanced compensatory functional coupling in individuals with ADS—arising from the need to maintain basic information transmission and network integration against a backdrop of increased early emotional and cognitive load—rather than a simple loss of network structure.

In contrast to the enhanced static connectivity, dynamic functional connectivity results revealed a decline in the ADS group’s ability to switch between brain network states. Previous studies on dynamic functional connectivity have shown that resting-state functional connectivity is not a fixed, static structure, but rather exhibits temporal variations and transient reorganization (20); flexible switching between functional states is believed to help maintain the brain’s adaptability to changes in internal and external demands. Based on this theoretical background, the present study found a reduction in the number of state transitions across all frequency bands in the ADS group, suggesting a weakened ability of their resting-state brain network to switch between different functional configurations. Although this shared reduction in dynamic agility mirrors the profound loss of network flexibility seen in clinically diagnosed adolescent MDD, the pathophysiological drivers are fundamentally distinct. In severe MDD, the loss of state-switching is generally the consequence of structuralized network fragmentation and a permanent “lock-in” to a pathological attractor state (21). In our subclinical ADS sample, however, this dynamic reduction must be interpreted in tandem with the aforementioned static hyper-coupling. From a macro-scale connectomics perspective, maintaining excessive static theta synchronization incurs a significant, continuous metabolic cost (22). Consequently, the dynamic rigidity in ADS likely reflects a state-dependent regulatory fatigue—a neural trade-off where the brain exhausts its dynamic switching capacity (adaptability) to sustain the essential, highly energy-demanding static hyper-synchronization (stability). Further analysis revealed that these dynamic abnormalities were primarily concentrated in the theta and beta frequency bands, manifesting as a reduction in transitions back to a baseline-like state and an increased probability of self-maintenance of specific states (23).

Abnormalities in state transitions within the beta frequency band may further reflect limitations in top-down regulatory processes in individuals with ADS. Beta oscillations are typically associated with the maintenance of current cognitive states, goal-directed processing, and top-down control. Therefore, the restricted state return and state persistence observed in the ADS group in the Beta band may indicate a decline in the dynamic regulatory capacity of their control-related networks (24). This finding may provide a potential neurodynamic explanation for the rumination, emotional rigidity, and difficulties with cognitive switching commonly observed in individuals with ADS. While severe clinical MDD is characterized by intractable ruminative loops driven by profound prefrontal deficits, the cognitive rigidity observed in our subclinical sample more likely represents an early, potentially reversible regulatory strain. However, since this study employed a cross-sectional design and the relationship between state transition measures and specific behavioral manifestations requires further validation, the above interpretation should be regarded as a mechanistic hypothesis specific to the early subclinical phase rather than a causal conclusion extrapolated to major depression.

It is worth noting that although the ADS group exhibited enhanced static coupling and reduced dynamic flexibility in the theta band, macroscopic higher-order topological measures did not show significant between-group differences after correction for multiple comparisons. The lack of significance in global TDA measures such as persistent entropy and landscape function may suggest the presence of a certain masking effect at the macroscopic whole-brain level: if network abnormalities associated with early depressive symptoms primarily occur in specific nodes, edges, or local subnetworks, whole-brain-level measures may average out these focal abnormalities along with compensatory connections in other regions, thereby weakening their statistical visibility (8, 9). Overall, the macroscopic-scale results suggest that the ADS brain network may be in an early abnormal state characterized by “enhanced static low-frequency coupling—reduced dynamic switching flexibility—relatively preserved global topology.” Therefore, it is necessary to further integrate analyses at the mesoscale (brain regions) and microscale (edges/nodes) levels to identify local vulnerable structures masked by global averaging effects and to provide a basis for more spatially specific candidate targets for subsequent intervention strategies, such as non-invasive brain stimulation.

### 4.3 Abnormalities in brain regions at the mesoscale: spatial masking effects and higher-order topological vulnerability

At the mesoanatomical scale, this study further clarified the spatial origins of macroscopic static over-coupling in the theta band. The results showed that the enhanced theta-band connectivity in the ADS group was not uniformly distributed throughout the brain but was primarily concentrated in a connectivity network centered on the central region, manifesting as enhanced functional connectivity within the central region as well as between the central region and the frontal, parietal, and temporal lobes. The central region primarily corresponds to the primary somatosensory and motor cortices, while the frontal and parietal lobes are involved in cognitive control, attentional allocation, and emotional regulation. Previous studies have shown that depressive disorders not only involve higher-order emotional and cognitive networks but may also manifest as imbalances among the somatosensory-motor network, the visual network, and the default mode network; depression in adolescents is also frequently accompanied by somatization symptoms or psychomotor changes (25, 26).When comparing individuals with depressive symptoms (ADS) and clinically diagnosed MDD populations, detailed network analyses reveal distinct stage-specific mechanisms of neural resource allocation. Extensive meta-analyses verify that severe clinical MDD is fundamentally characterized by widespread functional decoupling (hypoconnectivity) between frontoparietal cognitive networks and sensorimotor regions. This decoupling represents a systemic failure in top-down regulation, frequently manifesting as psychomotor retardation and somatic numbness. In our ADS cohort, we identified the exact opposite pattern—a localized hyper-connectivity. Therefore, rather than indicating network breakdown, the excessive theta coupling between the central and frontoparietal regions observed in this study reflects a highly demanding compensatory effort (27).

Unlike enhanced static connectivity, dynamic functional connectivity abnormalities exhibit more pronounced spatial and frequency-band specificity at the mesoscale. This study observed enhanced fluctuations in intra-frontal connectivity only in the delta band, suggesting that the prefrontal system in the ADS group may exhibit instability at the level of low-frequency dynamic regulation. The prefrontal cortex is a critical region for cognitive control, emotional regulation, and goal-directed behavior; recent intracranial EEG studies have also shown that the severity of depressive symptoms is associated with enhanced directional Delta-band connectivity between prefrontal subregions, suggesting that low-frequency oscillations in the prefrontal cortex may be involved in negative emotions and impaired emotional regulation (28). This finding identifies the prefrontal delta band as a shared vulnerable hub across the depression spectrum. However, a detailed breakdown of the dynamic temporal properties reveals diverging underlying mechanisms. In the severe clinical MDD patients examined by Myers et al., the enhanced delta connectivity presented as a fixed, trait-like structuralized pattern. Such static rigidity indicates that prefrontal regulatory circuits have lost adaptive responsiveness and are permanently locked into a pathological state. In our ADS group, this prefrontal delta network instead exhibited enhanced dynamic temporal fluctuations (variance). Because delta-band PLI estimation within 2-s windows involves a limited number of oscillatory cycles, this finding should be interpreted cautiously. Nevertheless, the frontal-frontal delta-band dFC abnormality was supported by two validation analyses: the Absolute SD validation confirmed that the effect was not solely driven by mean-normalized CV or baseline mean connectivity, and the mesoscale permutation–bootstrapping validation showed that the result remained stable under family-wise correction. Therefore, this finding is unlikely to be explained solely by normalization effects, multiple-comparison false positives, or a general short-window estimation artifact, although future studies should further validate it using longer or frequency-adaptive windows. Within this cautious methodological context, evaluated through the allostatic load framework (29) and dynamic connectomics, such high dynamic instability is a recognized biological signature of early allostatic strain. Physically, this variance represents a continuous, taxing neuro-regulatory “tug-of-war”—the prefrontal system is repeatedly attempting to mount top-down modulation against emerging depressive stressors, temporarily disengaging, and re-engaging. Therefore, the enhanced dynamic fluctuations in the Delta-band prefrontal connectivity in the ADS group may be one of the key mesoscale sources contributing to their reduced flexibility in state transitions at the macroscopic level. This dynamic variance objectively demonstrates that the prefrontal network in individuals with ADS is undergoing an active, ongoing adaptative struggle, which differs fundamentally from the exhausted, structuralized network collapse seen in severe depression. At the same time, reduced alpha-band connectivity within the occipital lobe and between the occipital and parietal lobes may indicate weakened processing of external visual information or diminished regulation of sensory input; given that alpha rhythms are typically associated with sensory inhibition, visual attention, and information gating, this finding can be interpreted as a possible manifestation of reduced involvement of external sensory processing in the resting-state brain network of individuals with ADS (30).Crucially, given that the current EEG data were acquired under an eyes-closed resting paradigm—a condition canonically defined by robust posterior alpha synchronization indicative of relaxed wakefulness—the focal alpha attenuation observed in the ADS group assumes specific pathophysiological significance. Beyond a mere deficit in passive sensory gating, this attenuation likely reflects a compromised capacity to initiate or sustain a normative neurophysiological idling state. Such an inability to disengage is plausibly underpinned by cortical hyperarousal and excessive internal rumination, which are central neurocognitive features of early depressive symptomatology.

It is worth noting that higher-order topological metrics at the mesoscale did not show significant between-group differences. Region-averaged node persistence did not reach statistical significance in any of the five major anatomical brain regions, suggesting that traditional lobar-level partitioning may be insufficient to capture more localized topological anomalies. This result does not necessarily imply that higher-order topological changes are absent in ADS; rather, it may reflect a spatial masking effect resulting from averaging across anatomical partitions. Brain network research has shown that node definitions, brain region partitioning methods, and spatial resolution significantly influence the reliability of functional connectivity and graph theory metrics; coarser anatomical partitioning may enhance overall stability but may also dilute local anomalies at the node or edge level (31). Therefore, if higher-order topological alterations in the ADS are primarily confined to a few key electrodes or local hubs, averaging at the lobar level may average out these anomalies along with relatively normal node activity within the same region, thereby weakening their statistical visibility.

Overall, the mesoscale results suggest that ADS brain network abnormalities may manifest as three complementary features: First, enhanced static coupling in the theta band centered on the central region, reflecting an active, high-demand compensatory integration between sensorimotor-related networks and cognitive control areas; Second, enhanced dynamic fluctuations in the delta band in the frontal lobe, suggesting an ongoing allostatic struggle (adaptive regulatory strain) in low-frequency modulatory processes in the prefrontal cortex; third, the absence of significant mesoscale higher-order topological indicators, suggesting that traditional spatial averaging at the lobar level may mask more localized topological abnormalities. Based on these findings, subsequent analyses should delve further down to the micro-electrode and edge levels to identify key nodes, abnormal edges, and potential candidates for neuromodulation that are masked by the averaging effect of brain regions.

### 4.4 Micro-scale edge and node anomalies: phase instability and topological distortions in core nodes

After overcoming the limitations of average effects at the macroscopic and mesoscopic levels, analysis at the microscopic sensor scale further revealed the local sources of brain network abnormalities in ADS. Following FDR multiple comparison correction at the whole-brain edge-level, significant abnormalities were primarily concentrated in the theta band, suggesting that theta-related networks may be a key frequency band for early brain functional abnormalities in ADS. Theta oscillations are closely associated with cognitive control, emotional regulation, working memory updating, and prefrontal-limbic system modulation; therefore, abnormalities in this frequency band may reflect changes in low-frequency synchrony regulation in individuals with ADS when emotional and cognitive loads increase (32).

At the static functional connectivity level, abnormal hyperconnectivity was primarily located along the longitudinal axis from the midline prefrontal cortex to the central region. Following rigorous statistical penalty, the Fz–FCz connection remained strictly significant, whereas the adjacent Fp1–FCz edge exhibited a trend-level effect. These results provide more refined, edge-level evidence for the enhanced Theta coupling in the central region observed at the mesoscale. The midline prefrontal regions are involved in self-related processing, emotional monitoring, and cognitive control, whereas the FCz and adjacent central regions are closely associated with sensorimotor integration, motor preparation, and action monitoring. Therefore, enhanced theta connectivity between the midline prefrontal cortex and the central region may reflect excessive monitoring of somatosensory, motor preparation, or interoceptive signals in individuals with ADS during rest. Rather than indicating a passive decoupling typical of severe depression, this edge-level excessive monitoring may be consistent with the “active compensatory integration” discussed earlier. The midline prefrontal cortex may therefore be involved in recruiting top-down control resources to integrate these early somatic disturbances. Nevertheless, given the scalp-level nature of EEG and the susceptibility of frontopolar and midline electrodes to residual ocular or myogenic activity, this interpretation should remain cautious. This interpretation is largely consistent with the view that depressive disorders involve psychomotor changes, somatization, and abnormalities in self-focused processing; however, it requires further validation in conjunction with behavioral and clinical symptoms (33, 34).

In contrast to static connectivity abnormalities, dynamic functional connectivity abnormalities primarily manifest as enhanced fluctuations in long-range connections from the right prefrontal cortex to the midline occipital visual areas, including Fp2–Oz and F4–Oz. The right prefrontal cortex is typically involved in sustained attention, emotional regulation, and top-down control, whereas the midline occipital visual areas are responsible for early visual processing. These findings suggest that the ADS group may exhibit instability in the prefrontal cortex’s dynamic regulation of visual sensory input. Previous studies have shown that the prefrontal cortex can modulate visual cortical activity through top-down mechanisms, and negative emotions and rumination may also be associated with abnormalities in visual imagery, endogenous image processing, and attentional control. Therefore, enhanced dynamic fluctuations in the right prefrontal-occipital theta network may reflect instability in the regulation of internal negative representations or visual-related information in individuals with ADS during rest (35, 36).Building upon the eyes-closed paradigm discussed earlier, the absence of external visual input implies that these dynamic fluctuations do not stem from environmental sensory tracking. Instead, they provide further compelling evidence for an ongoing neuro-regulatory “tug-of-war” over endogenously generated negative visual imagery and ruminative processes. Unlike the fixed loss of top-down control seen in severe MDD, these high-variance dynamic fluctuations indicate that the right prefrontal cortex is continuously, albeit unstably, attempting to suppress internal negative representations.

Higher-order topological analysis further revealed that right prefrontal nodes, particularly Fp2 and F4, exhibited reduced node frequency and node persistence in the theta band. Sustained synchrony and node persistence characterize the stable contributions of local nodes to multiscale connectivity from a network geometric perspective; thus, the decline in topological metrics for Fp2 and F4 suggests that the local topological support for the right prefrontal cortex as a hub for higher-order information integration may be weakened. Crucially, this localized topological fragility provides a mechanistic complement to the dynamic findings. Because Fp2 and F4 are the exact endpoints of the hyperactive dynamic pathways mentioned above, their reduced persistence is unlikely to represent irreversible structural degradation. Viewed through the lens of allostatic load, this decline more likely reflects a state of nodal fatigue—a direct consequence of sustaining the high-energy dynamic “tug-of-war” against internal negative rumination. Therefore, microscopic abnormalities in ADS do not manifest as isolated edge disruptions, but rather as a coupled phenomenon: intense compensatory connectivity fluctuations inevitably lead to higher-order topological fragility within the right prefrontal control system (37).

Furthermore, the local topological enhancement observed in the left temporal lobe FT7 node in the alpha band may suggest compensatory activity in language-, auditory-, or semantics-related networks. The left fronto-temporal region is typically involved in language processing and semantic integration, while alpha activity is associated with functional inhibition, information gating, and the modulation of regional processing states. Therefore, the topological enhancement at the FT7 node can be interpreted as a form of local compensation or abnormal engagement; however, given the relatively isolated nature of this finding, it should be treated as an exploratory observation (38).

Overall, the micro-scale results suggest that ADS brain network abnormalities exhibit distinct local clustering characteristics: static connectivity abnormalities are primarily concentrated along the midline prefrontal-central axis (acting as a compensatory somatic-cognitive bridge), dynamic connectivity abnormalities mainly involve the right prefrontal-visual pathway (reflecting an ongoing allostatic tug-of-war against negative imagery), and higher-order topological abnormalities further converge on nodes in the right prefrontal cortex (indicating localized overload and nodal fatigue). These results support the spatial masking effect proposed earlier, namely that abnormalities at the local node or edge level may be diluted in macroscopic whole-brain or mesoscale regional averages. Therefore, micro-electrode and edge-level analyses help identify more specific vulnerable nodes and abnormal pathways, and provide exploratory clues for more spatially specific candidate nodes for future intervention-related studies, such as non-invasive brain stimulation.

### 4.5 Multidimensional integration of evidence for exploratory scalp-level hubs and their translational implications

After identifying edge-level and node-level abnormalities in the theta band at the microscopic scale, this study further constructed a whole-brain multidimensional matrix of abnormal features to explore the spatial correspondence between data-driven brain network metrics and candidate neuromodulation targets. It is worth noting that the results at the edge and node levels show certain differences: after FDR multiple comparison correction, only a small number of edges remained significant for static functional connectivity abnormalities, whereas node-level metrics identified more abnormal hubs. These results are not contradictory. In graph theory, node degree or node strength inherently integrates connection information between a given node and other nodes throughout the brain; therefore, it better reflects the overall load of local hubs in global information integration than individual edges alone. Previous research in network neuroscience has also indicated that highly connected hubs play a critical role in whole-brain communication, functional integration, and system vulnerability; therefore, the finding of “sparse edges but significant nodes” may suggest that early abnormalities in ADS are not limited to single, isolated pathways, but are more likely to manifest as an accumulation of allostatic load at key hubs, structurally reflecting their systemic role in global compensatory efforts (39).

Within this node-hub-dominated framework, the multidimensional anomaly feature matrix reveals that several prefrontal electrode locations—including Fp1, Fp2, F3, Fz, and F4—exhibit high spatial overlap with prefrontal NIBS stimulation regions commonly used in existing clinical and research studies. It is important to emphasize that this study did not directly implement NIBS interventions; therefore, this spatial overlap should be interpreted as exploratory scalp-level computational evidence for future target investigation rather than as validation of therapeutic efficacy. Importantly, we must acknowledge the inherent anatomical uncertainty in this spatial mapping. The 10–20 EEG electrode positions represent approximate scalp-surface projections rather than precise point-to-point cortical targets. Actual clinical rTMS localization relies on MRI-guided neuronavigation to account for individual neuroanatomical variances. Therefore, scalp-electrode overlap should not be equated with impairment of a specific cortical stimulation target. The spatial overlap observed in our study should be strictly interpreted as scalp-level computational clues rather than definitive clinical stimulation coordinates. These scalp-level overlaps should also be interpreted with caution because residual artifacts may still affect frontopolar and midline electrodes, and future studies are needed to further validate these findings. Existing research indicates that the prefrontal cortex, particularly the dorsolateral prefrontal region, is the most commonly targeted area in rTMS and tDCS studies of depression; simultaneously, target selection based on individual functional connectivity is gradually emerging as an important approach to enhancing the precision of neurostimulation for depression (40). In this study, left and midline prefrontal nodes predominantly exhibited static connectivity abnormalities, whereas right prefrontal nodes Fp2 and F4 primarily exhibited abnormalities in two related topological indices, namely node frequency and node persistence. Given the strong correlation between NF and NP at these nodes, these results suggest that different prefrontal targets may correspond to different network-abnormality profiles: left and midline nodes were mainly associated with static connectivity alterations, whereas right prefrontal nodes were more prominently characterized by related higher-order topological abnormalities indexed by node frequency and node persistence.

In addition to traditional prefrontal candidate regions, this study also identified several non-traditional nodes that may have translational significance. First, the FCz exhibited prominent static connectivity abnormalities in the theta band. Combined with the edge-level results discussed earlier, the FCz may be a key node in the abnormal coupling axis between the midline prefrontal cortex and the central region, suggesting that sensorimotor-related networks may be involved in the somatization experiences, motor preparation, or psychomotor changes associated with ADS. Previous studies have shown that depressive disorders may be accompanied by abnormalities in sensorimotor networks and psychomotor symptoms, while motor and sensorimotor-related networks are also believed to be associated with emotional regulation and the phenotype of depressive symptoms. Therefore, acting as a central anchor for the “somatic-cognitive” compensatory bridge, the FCz and its adjacent central region networks can be regarded as candidate regulatory regions worthy of attention in future studies, rather than clinical targets that can be confirmed at this stage (41).

Second, the Oz exhibits relatively isolated abnormalities in dynamic functional connectivity metrics, suggesting that specific dynamic regulatory instability may exist in the visual midline region. The occipital visual cortex is not typically considered a first-line target for non-invasive brain stimulation (NIBS) in depression; however, abnormalities in visual processing, negative bias, and endogenous imagery processing have been linked to cognitive symptoms of depression. Consequently, the Oz abnormality may indicate alterations in visual information processing or internal representational regulation in individuals with ADS during rest. This finding is better suited as a clue for exploring mechanisms and phenotypic stratification rather than being directly interpreted as an independent therapeutic target (42).

Furthermore, topological anomalies in the FT7 node of the alpha band suggest that the left temporofrontal region may be involved in abnormalities in language, auditory, or semantic-emotional processing in ADS (43). Since FT7 is adjacent to language-related regions in the left frontotemporal area, its topological changes may reflect the involvement of local networks in semantic processing, inner speech, or emotional language processing in adolescent depression. As this finding is currently relatively isolated, it is best considered an exploratory finding. Future studies could further validate its functional significance by combining language tasks, auditory emotional tasks, or scales related to inner speech.

Overall, the multidimensional matrix of abnormal features establishes an exploratory link between ADS-associated network abnormalities and the approximate scalp projections of potential neuromodulation sites. Its primary value lies not in replacing existing clinical targets, but in suggesting that different nodes may correspond to different network-abnormality profiles: prefrontal nodes are more closely associated with static integration abnormalities or related higher-order topological vulnerability; the FCz may be associated with the sensorimotor–somatization profile; the Oz may be associated with visual information regulation; and the FT7 may be associated with language/semantic-emotional processing. Consequently, future studies on personalized neuromodulation for adolescent depressive symptoms may consider integrating patient-specific multidimensional network phenotypes to further explore target selection strategies based on computational models and network topological features.

## 5 Conclusions

This study established a multiscale brain network analysis framework that integrates adaptive thresholding, static/dynamic functional connectivity, and higher-order topological features to characterize abnormalities in the functional brain networks of adolescents with depressive symptoms (ADS). The results suggest that brain network alterations in ADS exhibit distinct spatial scale dependencies: at the macro- and meso-scales, these primarily manifest as enhanced static coupling in the theta band and reduced flexibility in dynamic state transitions, while at the micro-scale, the abnormalities appear to be concentrated in the midline prefrontal–central region connection, the right prefrontal–occipital dynamic pathway, and the higher-order topological vulnerability of right prefrontal nodes. These findings suggest that brain network abnormalities associated with adolescent depressive symptoms may not represent simple global network disruption, but rather an early pattern of network reorganization characterized by the coexistence of locally vulnerable nodes, abnormal connections, and systemic compensation. Additionally, the multidimensional matrix of abnormal features provides network-level insights into the approximate scalp projections of prefrontal neuromodulation sites and suggests that non-traditional nodes such as FCz, Oz, and FT7 may warrant further investigation.

It should be noted that this study has several limitations. First, the cross-sectional design precludes conclusions about the causal sequence of EEG network abnormalities in adolescents with depressive symptoms. Second, although scalp EEG provides high temporal resolution, its spatial localization accuracy is limited. Therefore, the present findings should be interpreted primarily as sensor-level network alterations rather than precise localization of underlying cortical or subcortical sources. Third, residual non-neural artifacts cannot be completely excluded. Although ICA-based correction, visual/manual inspection, amplitude-based rejection, and PLI connectivity were used to reduce ocular, myogenic, and volume-conduction-related confounds, findings involving frontopolar and midline electrodes such as Fp1, Fp2, Fz, FCz, and Oz should still be interpreted cautiously. Fourth, although the 2-s time window improved the temporal resolution of the dynamic analysis, it may have affected the stability of low-frequency phase estimation. This issue is particularly relevant to the delta band, for which each 2-s window contained only approximately 2–8 oscillatory cycles, compared with approximately 8–14 cycles in the theta band, 16–26 cycles in the alpha band, and 28–60 cycles in the beta band. Short-window phase-based connectivity estimation may reduce the stability of PLI estimates and increase their variance, especially in the delta band, thereby potentially inflating the variability of windowed dFC measures. Although the delta-band frontal dFC abnormality was supported by the Absolute SD validation and permutation–bootstrapping validation, it should still be interpreted cautiously as a marker of low-frequency temporal instability rather than as a precise estimate of absolute phase-locking strength. Future studies should validate low-frequency connectivity findings using longer or frequency-adaptive windows. Fifth, the clinical interpretation and diagnostic specificity of the present findings are limited by the symptom-defined, scale-based, and two-group case-control design. The ADS group was defined using CES-D and PHQ-9 threshold scores combined with review by school counseling professionals; however, no structured diagnostic interview such as SCID or MINI was administered. Moreover, the CES-D and PHQ-9 are both self-report measures of depressive symptoms and assess closely related constructs. Thus, the dual-threshold criterion should be interpreted as a conservative screening procedure requiring consistent symptom elevation across two validated scales, rather than as evidence that the two scales captured independent symptom dimensions or unique variance beyond the shared depressive-symptom construct. Under this sampling framework, the present findings should be interpreted as EEG network correlates of elevated depressive symptoms in adolescents, rather than diagnostic biomarkers or definitive mechanisms of clinically diagnosed major depressive disorder. Comparisons with studies of clinically diagnosed MDD should therefore be understood as contextual comparisons for interpreting possible similarities and differences between symptom-defined ADS and more severe depressive disorders, not as evidence of diagnostic equivalence. In addition, because the present study included only ADS and HC groups and did not include psychiatric clinical control groups, such as adolescents with anxiety disorders, externalizing disorders, or other forms of psychopathology, we cannot determine whether the observed EEG network alterations are specific to depressive symptoms or reflect broader transdiagnostic neural correlates of psychopathology. Finally, because participants were selected using threshold-based ADS and HC criteria rather than a continuous symptom-spectrum design, the present study was not designed to test dimensional symptom–network associations. The compensatory versus maladaptive interpretation of theta-band hyperconnectivity should therefore be regarded as hypothesis-generating. Finally, the mapping of regulatory hubs remains an exploratory computational inference. Because 10–20 scalp EEG electrodes cannot be mapped directly onto individualized cortical stimulation targets without MRI-guided neuronavigation, the observed spatial overlap should not be interpreted as validated neuromodulation targets. Future research should integrate structured diagnostic interviews, clinically diagnosed MDD samples, continuous symptom-spectrum cohorts with dimensional symptom–network modeling, more detailed artifact quantification, EOG-informed correction, alternative referencing schemes, multicenter longitudinal designs, high-density EEG source localization, EEG-fMRI multimodal data, and large-sample randomized controlled intervention trials to further validate the neural origin, stability, developmental specificity, and clinical translational value of these network features, as well as their potential application in personalized neurostimulation target selection and treatment-response prediction.

## Data Availability

The raw data supporting the conclusions of this article will be made available by the authors, without undue reservation.
