# Supplementary material for: Multiscale EEG network alterations in adolescents with depressive symptoms: static–dynamic connectivity, topological features, and exploratory scalp-level network hubs
Source: Front Psychiatry. 2026 Oct 26;17:1943613. doi: 10.3389/fpsyt.2026.1943613 (PMC13746005; doi:10.3389/fpsyt.2026.1943613)
Supplement: Supplementary file 1 [file Table1.docx]

Table S1:Inter-group differences in global static functional connectivity (sFC) between the HC and ADS groups across four frequency bands

| **Frequency Band** | **HC**  **(n=154, Mean ± SD)** | **ADS**  **(n=96, Mean ± SD)** | ***U*** | ***r*** | ***P*** | ***P*_FDR_** | **Sig.** |
| --- | --- | --- | --- | --- | --- | --- | --- |
| Delta | 0.227 ± 0.011 | 0.227 ± 0.010 | 7381.5 | 0.001 | 0.986 | 0.986 | ns |
| Theta | 0.181 ± 0.007 | 0.189 ± 0.017 | 5243.5 | 0.291 | <0.001 | <0.001 | *** |
| Alpha | 0.234 ± 0.044 | 0.230 ± 0.050 | 7967.5 | -0.078 | 0.301 | 0.402 | ns |
| Beta | 0.104 ± 0.025 | 0.112 ± 0.034 | 6195.5 | 0.162 | 0.031 | 0.063 | ns |

Note: Data are presented as Mean ± SD. HC = Healthy Controls; ADS = Adolescents with depressive symptoms; *U* = Mann-Whitney U statistic; *r* = effect size; *P* = uncorrected P-value; *P*_FDR_= P-value corrected using the False Discovery Rate method. * indicates *P*_FDR_ < 0.05, **indicates *P*_FDR_ < 0.01, *** indicates *P*_FDR_< 0.001; ns = not significant.

Table S2: Inter-group differences in global dynamic functional connectivity (dFC) features between the HC and ADS groups across four frequency bands

| **Frequency Band** | **Metrics** | **HC**  **(n=154, Mean ± SD)** | **ADS**  **(n=96, Mean ± SD)** | ***U*** | ***r*** | ***P*** | ***P_FDR_*** | ***Sig.*** |
| --- | --- | --- | --- | --- | --- | --- | --- | --- |
| Delta | Fractional Time S1 | 0.583 ± 0.058 | 0.578 ± 0.055 | 7839.5 | -0.061 | 0.421 | 0.421 | ns |
|  | Fractional Time S2 | 0.228 ± 0.045 | 0.222 ± 0.056 | 7846.5 | -0.061 | 0.414 | 0.512 | ns |
|  | Fractional Time S3 | 0.189 ± 0.047 | 0.200 ± 0.051 | 6343.5 | 0.142 | 0.059 | 0.119 | ns |
|  | Total Transitions | 66.903 ± 17.366 | 60.958 ± 20.957 | 8601.5 | -0.164 | 0.030 | 0.030 | * |
|  | Transition S1 → S1 | 0.585 ± 0.074 | 0.575 ± 0.074 | 8094.5 | -0.095 | 0.207 | 0.207 | ns |
|  | Transition S1 → S2 | 0.226 ± 0.063 | 0.221 ± 0.062 | 7722.5 | -0.045 | 0.553 | 0.553 | ns |
|  | Transition S1 → S3 | 0.189 ± 0.062 | 0.205 ± 0.060 | 6087 | 0.177 | 0.019 | 0.076 | ns |
|  | Transition S2 → S1 | 0.585 ± 0.107 | 0.577 ± 0.113 | 7851.5 | -0.062 | 0.409 | 0.545 | ns |
|  | Transition S2 → S2 | 0.224 ± 0.087 | 0.221 ± 0.098 | 7443 | -0.007 | 0.928 | 0.928 | ns |
|  | Transition S2 → S3 | 0.191 ± 0.083 | 0.201 ± 0.106 | 7201 | 0.026 | 0.732 | 0.976 | ns |
|  | Transition S3 → S1 | 0.576 ± 0.117 | 0.574 ± 0.122 | 7370 | 0.003 | 0.969 | 0.969 | ns |
|  | Transition S3 → S2 | 0.237 ± 0.101 | 0.234 ± 0.121 | 7659.5 | -0.036 | 0.631 | 0.737 | ns |
|  | Transition S3 → S3 | 0.187 ± 0.088 | 0.192 ± 0.097 | 7114 | 0.038 | 0.618 | 0.824 | ns |

Table S2 (continued)

| **Frequency Band** | **Metrics** | **HC**  **(n=154, Mean ± SD)** | **ADS**  **(n=96, Mean ± SD)** | ***U*** | ***r*** | ***P*** | ***P_FDR_*** | ***Sig.*** |
| --- | --- | --- | --- | --- | --- | --- | --- | --- |
| Theta | Fractional Time S1 | 0.632 ± 0.053 | 0.597 ± 0.108 | 8711 | -0.178 | 0.018 | 0.035 | * |
|  | Fractional Time S2 | 0.183 ± 0.048 | 0.213 ± 0.111 | 6003 | 0.188 | 0.013 | 0.050 | ns |
|  | Fractional Time S3 | 0.185 ± 0.045 | 0.190 ± 0.064 | 7279.5 | 0.015 | 0.84 | 0.840 | ns |
|  | Total Transitions | 63.065 ± 16.470 | 57.172 ± 20.033 | 8628 | -0.167 | 0.026 | 0.030 | * |
|  | Transition S1 → S1 | 0.626 ± 0.063 | 0.596 ± 0.117 | 8363.5 | -0.131 | 0.081 | 0.162 | ns |
|  | Transition S1 → S2 | 0.184 ± 0.054 | 0.203 ± 0.084 | 6356 | 0.14 | 0.063 | 0.206 | ns |
|  | Transition S1 → S3 | 0.190 ± 0.051 | 0.191 ± 0.077 | 7605.5 | -0.029 | 0.702 | 0.702 | ns |
|  | Transition S2 → S1 | 0.639 ± 0.119 | 0.596 ± 0.152 | 8627.5 | -0.167 | 0.026 | 0.053 | ns |
|  | Transition S2 → S2 | 0.181 ± 0.085 | 0.213 ± 0.142 | 6582.5 | 0.11 | 0.146 | 0.291 | ns |
|  | Transition S2 → S3 | 0.174 ± 0.093 | 0.191 ± 0.114 | 7071 | 0.043 | 0.564 | 0.976 | ns |
|  | Transition S3 → S1 | 0.638 ± 0.115 | 0.600 ± 0.159 | 8254 | -0.117 | 0.121 | 0.485 | ns |
|  | Transition S3 → S2 | 0.184 ± 0.104 | 0.201 ± 0.131 | 6821.5 | 0.077 | 0.305 | 0.61 | ns |
|  | Transition S3 → S3 | 0.178 ± 0.088 | 0.199 ± 0.109 | 6551 | 0.114 | 0.131 | 0.261 | ns |
| Alpha | Fractional Time S1 | 0.618 ± 0.160 | 0.637 ± 0.166 | 6800.5 | 0.08 | 0.288 | 0.384 | ns |
|  | Fractional Time S2 | 0.213 ± 0.095 | 0.202 ± 0.088 | 7757 | -0.049 | 0.512 | 0.512 | ns |
|  | Fractional Time S3 | 0.169 ± 0.130 | 0.160 ± 0.128 | 7733 | -0.046 | 0.540 | 0.720 | ns |
|  | Total Transitions | 57.218 ± 21.667 | 48.307 ± 21.197 | 9131 | -0.235 | 0.002 | 0.007 | ** |
|  | Transition S1 → S1 | 0.636 ± 0.153 | 0.659 ± 0.164 | 6631 | 0.103 | 0.171 | 0.207 | ns |
|  | Transition S1 → S2 | 0.205 ± 0.089 | 0.195 ± 0.094 | 7934 | -0.073 | 0.330 | 0.440 | ns |
|  | Transition S1 → S3 | 0.159 ± 0.126 | 0.146 ± 0.120 | 7998.5 | -0.082 | 0.276 | 0.368 | ns |
|  | Transition S2 → S1 | 0.607 ± 0.185 | 0.610 ± 0.215 | 7258.5 | 0.018 | 0.811 | 0.811 | ns |
|  | Transition S2 → S2 | 0.223 ± 0.127 | 0.209 ± 0.137 | 7775.5 | -0.052 | 0.491 | 0.654 | ns |
|  | Transition S2 → S3 | 0.171 ± 0.146 | 0.181 ± 0.175 | 7405.5 | -0.002 | 0.981 | 0.981 | ns |

Table S2 (continued)

| **Frequency Band** | **Metrics** | **HC**  **(n=154, Mean ± SD)** | **ADS**  **(n=96, Mean ± SD)** | ***U*** | ***r*** | ***P*** | ***P_FDR_*** | ***Sig.*** |
| --- | --- | --- | --- | --- | --- | --- | --- | --- |
| Alpha | Transition S3 → S1 | 0.593 ± 0.219 | 0.583 ± 0.229 | 7575 | -0.025 | 0.743 | 0.969 | ns |
|  | Transition S3 → S2 | 0.211 ± 0.153 | 0.212 ± 0.147 | 7205 | 0.025 | 0.737 | 0.737 | ns |
|  | Transition S3 → S3 | 0.196 ± 0.158 | 0.194 ± 0.162 | 7484.5 | -0.013 | 0.868 | 0.868 | ns |
| Beta | Fractional Time S1 | 0.671 ± 0.127 | 0.601 ± 0.189 | 9036 | -0.222 | 0.003 | 0.012 | * |
|  | Fractional Time S2 | 0.303 ± 0.078 | 0.343 ± 0.130 | 6169 | 0.165 | 0.028 | 0.056 | ns |
|  | Fractional Time S3 | 0.026 ± 0.103 | 0.056 ± 0.159 | 5842 | 0.21 | 0.003 | 0.013 | * |
|  | Total Transitions | 49.773 ± 15.299 | 45.260 ± 17.316 | 8698 | -0.177 | 0.019 | 0.030 | * |
|  | Transition S1 → S1 | 0.679 ± 0.110 | 0.622 ± 0.174 | 8739.5 | -0.182 | 0.015 | 0.062 | ns |
|  | Transition S1 → S2 | 0.303 ± 0.090 | 0.333 ± 0.142 | 6484.5 | 0.123 | 0.103 | 0.206 | ns |
|  | Transition S1 → S3 | 0.011 ± 0.023 | 0.024 ± 0.103 | 6836 | 0.075 | 0.269 | 0.368 | ns |
|  | Transition S2 → S1 | 0.668 ± 0.143 | 0.590 ± 0.201 | 9126 | -0.235 | 0.002 | 0.007 | ** |
|  | Transition S2 → S2 | 0.310 ± 0.101 | 0.361 ± 0.146 | 5848 | 0.209 | 0.006 | 0.022 | * |
|  | Transition S2 → S3 | 0.022 ± 0.085 | 0.038 ± 0.098 | 6117.5 | 0.172 | 0.005 | 0.020 | * |
|  | Transition S3 → S1 | 0.287 ± 0.414 | 0.253 ± 0.367 | 7331 | 0.008 | 0.902 | 0.969 | ns |
|  | Transition S3 → S2 | 0.189 ± 0.343 | 0.256 ± 0.346 | 6354.5 | 0.14 | 0.028 | 0.112 | ns |
|  | Transition S3 → S3 | 0.031 ± 0.135 | 0.085 ± 0.226 | 6667.5 | 0.098 | 0.011 | 0.043 | * |

Note. Data are presented as mean ± SD. HC = healthy controls; ADS = adolescents with depressive symptoms. Fractional Time indicates the proportion of total time spent in a specific dFCS state. Total Transitions indicates the total number of transitions between dFCS states. Transition Sx→Sy indicates the Markov transition probability from dFCS state x to dFCS state y. *U* = Mann-Whitney U statistic; *r* = effect size; *P* = uncorrected P-value; *P*_FDR_= P-value corrected using the False Discovery Rate method. * indicates *P*_FDR_ < 0.05, **indicates *P*_FDR_ < 0.01, *** indicates *P*_FDR_< 0.001; ns = not significant.

Table S3. Inter-group differences in global higher-order topological features (TDA) between the HC and ADS groups across four frequency bands

| **Frequency Band** | **Metrics** | **HC**  **(n=154, Mean ± SD)** | **ADS**  **(n=96, Mean ± SD)** | ***U*** | ***r*** | ***P*** | ***P_FDR_*** | ***Sig.*** |
| --- | --- | --- | --- | --- | --- | --- | --- | --- |
| Delta | H1 Entropy | 1.346 ± 1.171 | 1.440 ± 1.134 | 7071 | 0.043 | 0.557 | 0.557 | ns |
|  | H1 L1 Norm | 0.000 ± 0.000 | 0.000 ± 0.000 | 6818 | 0.078 | 0.297 | 0.594 | ns |
|  | H1 L2 Norm | 0.001 ± 0.001 | 0.001 ± 0.001 | 6889 | 0.068 | 0.361 | 0.721 | ns |
| Theta | H1 Entropy | 1.474 ± 1.164 | 1.118 ± 1.117 | 8728.5 | -0.181 | 0.014 | 0.057 | ns |
|  | H1 L1 Norm | 0.000 ± 0.000 | 0.000 ± 0.001 | 7663.5 | -0.037 | 0.623 | 0.83 | ns |
|  | H1 L2 Norm | 0.001 ± 0.001 | 0.001 ± 0.002 | 7683.5 | -0.039 | 0.597 | 0.796 | ns |
| Alpha | H1 Entropy | 1.887 ± 1.074 | 2.066 ± 1.100 | 6611 | 0.106 | 0.16 | 0.320 | ns |
|  | H1 L1 Norm | 0.001 ± 0.002 | 0.001 ± 0.002 | 7425.5 | -0.005 | 0.953 | 0.953 | ns |
|  | H1 L2 Norm | 0.003 ± 0.004 | 0.003 ± 0.004 | 7425.5 | -0.005 | 0.953 | 0.953 | ns |
| Beta | H1 Entropy | 1.761 ± 1.119 | 1.869 ± 1.104 | 7026.5 | 0.049 | 0.51 | 0.557 | ns |
|  | H1 L1 Norm | 0.000 ± 0.001 | 0.000 ± 0.001 | 6089.5 | 0.176 | 0.019 | 0.076 | ns |
|  | H1 L2 Norm | 0.001 ± 0.002 | 0.001 ± 0.003 | 6117.5 | 0.172 | 0.022 | 0.087 | ns |

Note;Data are presented as Mean ± SD. HC = Healthy Controls; ADS = Adolescents with depressive symptoms; Persistent Entropy = 1D persistent entropy, quantifying the global complexity of topological loops in the brain network; Landscape L1-Norm / L2-Norm = the L1 and L2 norms of the 1D persistent landscape, reflecting the overall robustness of higher-order topological structures; *U* = Mann-Whitney U statistic; *r* = effect size; *P* = uncorrected P-value; *P*_FDR_= P-value corrected using the False Discovery Rate method. * indicates *P*_FDR_ < 0.05, **indicates *P*_FDR_ < 0.01, *** indicates *P*_FDR_< 0.001; ns = not significant.

Table S4. Inter-group differences in mesoscale static functional connectivity (sFC) between the HC and ADS groups across four frequency bands

| **Frequency Band** | **Connection (Edge)** | **HC**  **(n=154, Mean ± SD)** | **ADS**  **(n=96, Mean ± SD)** | ***U*** | ***r*** | ***P*** | ***P_FDR_*** | ***Sig.*** |
| --- | --- | --- | --- | --- | --- | --- | --- | --- |
| Delta | Central - Central | 0.222 ± 0.016 | 0.223 ± 0.017 | 7603.5 | 0.029 | 0.704 | 0.820 | ns |
|  | Central - Occipital | 0.227 ± 0.020 | 0.226 ± 0.021 | 7178.5 | -0.029 | 0.702 | 0.820 | ns |
|  | Central - Parietal | 0.225 ± 0.017 | 0.226 ± 0.016 | 7598.5 | 0.028 | 0.711 | 0.820 | ns |
|  | Central - Temporal | 0.227 ± 0.016 | 0.227 ± 0.016 | 7352.5 | -0.005 | 0.944 | 0.960 | ns |
|  | Frontal - Central | 0.227 ± 0.014 | 0.226 ± 0.015 | 6772 | -0.084 | 0.265 | 0.568 | ns |
|  | Frontal - Frontal | 0.229 ± 0.018 | 0.227 ± 0.022 | 6875.5 | -0.07 | 0.353 | 0.643 | ns |
|  | Frontal - Occipital | 0.230 ± 0.020 | 0.231 ± 0.021 | 7446.5 | 0.007 | 0.923 | 0.954 | ns |
|  | Frontal - Parietal | 0.228 ± 0.017 | 0.229 ± 0.016 | 7887.5 | 0.067 | 0.373 | 0.643 | ns |
|  | Frontal - Temporal | 0.228 ± 0.017 | 0.229 ± 0.017 | 7791.5 | 0.054 | 0.473 | 0.692 | ns |
|  | Occipital - Occipital | 0.228 ± 0.034 | 0.225 ± 0.035 | 6926.5 | -0.063 | 0.403 | 0.654 | ns |
|  | Parietal - Occipital | 0.227 ± 0.023 | 0.225 ± 0.023 | 7267.5 | -0.017 | 0.824 | 0.885 | ns |
|  | Parietal - Parietal | 0.225 ± 0.023 | 0.226 ± 0.027 | 7539.5 | 0.02 | 0.792 | 0.879 | ns |
|  | Temporal - Occipital | 0.231 ± 0.022 | 0.229 ± 0.022 | 7182.5 | -0.028 | 0.707 | 0.820 | ns |
|  | Temporal - Parietal | 0.229 ± 0.019 | 0.230 ± 0.017 | 7965.5 | 0.078 | 0.303 | 0.606 | ns |
|  | Temporal - Temporal | 0.230 ± 0.022 | 0.230 ± 0.021 | 7482.5 | 0.012 | 0.871 | 0.917 | ns |
| Theta | Central - Central | 0.178 ± 0.013 | 0.190 ± 0.026 | 9581.5 | 0.296 | <0.001 | 0.002 | ** |
|  | Central - Occipital | 0.188 ± 0.019 | 0.190 ± 0.026 | 7126.5 | -0.036 | 0.634 | 0.820 | ns |
|  | Central - Parietal | 0.179 ± 0.011 | 0.190 ± 0.027 | 9470.5 | 0.281 | <0.001 | 0.004 | ** |
|  | Central - Temporal | 0.180 ± 0.012 | 0.190 ± 0.026 | 9132.5 | 0.235 | 0.002 | 0.018 | * |
|  | Frontal - Central | 0.178 ± 0.013 | 0.191 ± 0.026 | 9715.5 | 0.314 | <0.001 | 0.002 | ** |
|  | Frontal - Frontal | 0.180 ± 0.016 | 0.189 ± 0.023 | 9261.5 | 0.253 | <0.001 | 0.012 | * |
|  | Frontal - Occipital | 0.191 ± 0.022 | 0.191 ± 0.028 | 6960.5 | -0.058 | 0.438 | 0.678 | ns |
|  | Frontal - Parietal | 0.180 ± 0.013 | 0.191 ± 0.033 | 8792.5 | 0.189 | 0.012 | 0.064 | ns |

Table S4 (continued)

| **Frequency Band** | | **Connection (Edge)** | **HC**  **(n=154, Mean ± SD)** | ADS  **(n=96, Mean ± SD)** | ***U*** | ***r*** | ***P*** | ***P_FDR_*** | ***Sig.*** |
| --- | --- | --- | --- | --- | --- | --- | --- | --- | --- |
| Theta | Frontal - Temporal | | 0.180 ± 0.014 | 0.189 ± 0.029 | 8626.5 | 0.167 | 0.026 | 0.099 | ns |
|  | Occipital - Occipital | | 0.187 ± 0.030 | 0.187 ± 0.035 | 7179.5 | -0.029 | 0.703 | 0.820 | ns |
|  | Parietal - Occipital | | 0.186 ± 0.018 | 0.192 ± 0.035 | 7821.5 | 0.058 | 0.440 | 0.678 | ns |
|  | Parietal - Parietal | | 0.180 ± 0.020 | 0.193 ± 0.039 | 8911.5 | 0.206 | 0.006 | 0.044 | * |
|  | Temporal - Occipital | | 0.188 ± 0.017 | 0.190 ± 0.027 | 7092.5 | -0.041 | 0.591 | 0.788 | ns |
|  | Temporal - Parietal | | 0.181 ± 0.013 | 0.193 ± 0.034 | 9090.5 | 0.230 | 0.002 | 0.019 | * |
|  | Temporal - Temporal | | 0.181 ± 0.018 | 0.191 ± 0.040 | 8702.5 | 0.177 | 0.018 | 0.085 | ns |
| Alpha | Central - Central | | 0.218 ± 0.044 | 0.223 ± 0.060 | 7381.5 | -0.001 | 0.986 | 0.986 | ns |
|  | Central - Occipital | | 0.258 ± 0.065 | 0.256 ± 0.083 | 6709.5 | -0.092 | 0.220 | 0.489 | ns |
|  | Central - Parietal | | 0.246 ± 0.057 | 0.244 ± 0.068 | 6909.5 | -0.065 | 0.386 | 0.643 | ns |
|  | Central - Temporal | | 0.214 ± 0.039 | 0.213 ± 0.048 | 7013.5 | -0.051 | 0.497 | 0.693 | ns |
|  | Frontal - Central | | 0.228 ± 0.055 | 0.231 ± 0.066 | 7269.5 | -0.017 | 0.826 | 0.885 | ns |
|  | Frontal - Frontal | | 0.203 ± 0.043 | 0.205 ± 0.041 | 7875.5 | 0.065 | 0.385 | 0.643 | ns |
|  | Frontal - Occipital | | 0.267 ± 0.078 | 0.251 ± 0.079 | 6325.5 | -0.144 | 0.055 | 0.166 | ns |
|  | Frontal - Parietal | | 0.257 ± 0.066 | 0.245 ± 0.067 | 6485.5 | -0.123 | 0.103 | 0.269 | ns |
|  | Frontal - Temporal | | 0.215 ± 0.049 | 0.210 ± 0.039 | 7184.5 | -0.028 | 0.710 | 0.820 | ns |
|  | Occipital - Occipital | | 0.270 ± 0.077 | 0.240 ± 0.064 | 5657.5 | -0.235 | 0.002 | 0.018 | * |
|  | Parietal - Occipital | | 0.266 ± 0.058 | 0.249 ± 0.055 | 5880.5 | -0.204 | 0.007 | 0.044 | * |
|  | Parietal - Parietal | | 0.246 ± 0.056 | 0.232 ± 0.051 | 6224 | -0.158 | 0.036 | 0.119 | ns |
|  | Temporal - Occipital | | 0.245 ± 0.054 | 0.237 ± 0.062 | 6429.5 | -0.13 | 0.084 | 0.239 | ns |
|  | Temporal - Parietal | | 0.239 ± 0.051 | 0.230 ± 0.051 | 6533.5 | -0.116 | 0.123 | 0.307 | ns |
|  | Temporal - Temporal | | 0.223 ± 0.052 | 0.213 ± 0.044 | 6804.5 | -0.079 | 0.291 | 0.602 | ns |

Table S4 (continued)

| **Frequency Band** | **Connection (Edge)** | **HC**  **(n=154, Mean ± SD)** | **ADS**  **(n=96, Mean ± SD)** | ***U*** | ***r*** | ***P*** | ***P_FDR_*** | ***Sig.*** |
| --- | --- | --- | --- | --- | --- | --- | --- | --- |
| Beta | Central - Central | 0.102 ± 0.024 | 0.114 ± 0.044 | 8755.5 | 0.184 | 0.014 | 0.071 | ns |
|  | Central - Occipital | 0.105 ± 0.030 | 0.111 ± 0.049 | 7805.5 | 0.056 | 0.458 | 0.687 | ns |
|  | Central - Parietal | 0.106 ± 0.024 | 0.115 ± 0.037 | 8647.5 | 0.17 | 0.024 | 0.097 | ns |
|  | Central - Temporal | 0.102 ± 0.026 | 0.112 ± 0.042 | 8873.5 | 0.2 | 0.008 | 0.046 | * |
|  | Frontal - Central | 0.102 ± 0.021 | 0.115 ± 0.044 | 8644.5 | 0.169 | 0.024 | 0.097 | ns |
|  | Frontal - Frontal | 0.101 ± 0.024 | 0.113 ± 0.044 | 8570.5 | 0.159 | 0.034 | 0.119 | ns |
|  | Frontal - Occipital | 0.102 ± 0.025 | 0.110 ± 0.055 | 7556.5 | 0.022 | 0.768 | 0.869 | ns |
|  | Frontal - Parietal | 0.105 ± 0.021 | 0.114 ± 0.035 | 8477.5 | 0.147 | 0.051 | 0.161 | ns |
|  | Frontal - Temporal | 0.101 ± 0.022 | 0.109 ± 0.038 | 8174.5 | 0.106 | 0.16 | 0.368 | ns |
|  | Occipital - Occipital | 0.111 ± 0.035 | 0.108 ± 0.043 | 6447.5 | -0.128 | 0.09 | 0.244 | ns |
|  | Parietal - Occipital | 0.109 ± 0.026 | 0.112 ± 0.040 | 6834.5 | -0.075 | 0.317 | 0.613 | ns |
|  | Parietal - Parietal | 0.108 ± 0.025 | 0.117 ± 0.043 | 7702.5 | 0.042 | 0.577 | 0.787 | ns |
|  | Temporal - Occipital | 0.104 ± 0.026 | 0.105 ± 0.039 | 6592.5 | -0.108 | 0.151 | 0.362 | ns |
|  | Temporal - Parietal | 0.105 ± 0.024 | 0.112 ± 0.035 | 7892.5 | 0.068 | 0.369 | 0.643 | ns |
|  | Temporal - Temporal | 0.102 ± 0.024 | 0.109 ± 0.043 | 7781.5 | 0.053 | 0.484 | 0.692 | ns |

Note. Data are presented as Mean ± SD. HC = Healthy Controls; ADS = Adolescents with depressive symptoms; Metrics denote the static phase-lag coupling strength between specific cortical regions; *U* = Mann-Whitney U statistic; *r* = effect size; *P* = uncorrected P-value; *P*_FDR_= P-value corrected using the False Discovery Rate method. * indicates *P*_FDR_ < 0.05, **indicates *P*_FDR_ < 0.01, *** indicates *P*_FDR_< 0.001; ns = not significant.

Table S5. Inter-group differences in mesoscale dynamic functional connectivity (dFC) between the HC and ADS groups across four frequency bands

| **Frequency Band** | **Connection (Edge)** | **HC**  **(n=154, Mean ± SD)** | **ADS**  **(n=96, Mean ± SD)** | ***U*** | ***r*** | ***P*** | ***P_FDR_*** | ***Sig.*** |
| --- | --- | --- | --- | --- | --- | --- | --- | --- |
| Delta | Central - Central | 0.509 ± 0.068 | 0.511 ± 0.071 | 7441.5 | 0.007 | 0.93 | 0.992 | ns |
|  | Central - Occipital | 0.642 ± 0.097 | 0.661 ± 0.118 | 7932.5 | 0.073 | 0.332 | 0.526 | ns |
|  | Central - Parietal | 0.483 ± 0.063 | 0.489 ± 0.069 | 7738.5 | 0.047 | 0.534 | 0.648 | ns |
|  | Central - Temporal | 0.500 ± 0.069 | 0.498 ± 0.078 | 7020.5 | -0.05 | 0.505 | 0.644 | ns |
|  | Frontal - Central | 0.541 ± 0.074 | 0.542 ± 0.088 | 7395.5 | 0 | 0.996 | 0.996 | ns |
|  | Frontal - Frontal | 0.591 ± 0.099 | 0.636 ± 0.105 | 9447.5 | 0.278 | <0.001 | 0.013 | * |
|  | Frontal - Occipital | 0.713 ± 0.123 | 0.757 ± 0.145 | 8839.5 | 0.196 | 0.009 | 0.1 | ns |
|  | Frontal - Parietal | 0.559 ± 0.091 | 0.580 ± 0.096 | 8556.5 | 0.158 | 0.036 | 0.143 | ns |
|  | Frontal - Temporal | 0.584 ± 0.093 | 0.588 ± 0.111 | 7196.5 | -0.026 | 0.726 | 0.806 | ns |
|  | Occipital - Occipital | 0.928 ± 0.191 | 0.970 ± 0.181 | 8354.5 | 0.13 | 0.084 | 0.227 | ns |
|  | Parietal - Occipital | 0.585 ± 0.106 | 0.611 ± 0.088 | 8610.5 | 0.165 | 0.029 | 0.124 | ns |
|  | Parietal - Parietal | 0.674 ± 0.113 | 0.670 ± 0.126 | 7065.5 | -0.044 | 0.558 | 0.648 | ns |
|  | Temporal - Occipital | 0.604 ± 0.104 | 0.638 ± 0.109 | 8616.5 | 0.166 | 0.028 | 0.124 | ns |
|  | Temporal - Parietal | 0.488 ± 0.078 | 0.486 ± 0.078 | 7068.5 | -0.044 | 0.561 | 0.648 | ns |
|  | Temporal - Temporal | 0.603 ± 0.092 | 0.579 ± 0.099 | 6441.5 | -0.129 | 0.088 | 0.227 | ns |
| Theta | Central - Central | 0.535 ± 0.078 | 0.517 ± 0.086 | 6451 | -0.127 | 0.091 | 0.227 | ns |
|  | Central - Occipital | 0.623 ± 0.103 | 0.648 ± 0.114 | 8264.5 | 0.118 | 0.117 | 0.27 | ns |
|  | Central - Parietal | 0.487 ± 0.065 | 0.479 ± 0.075 | 6853.5 | -0.073 | 0.333 | 0.526 | ns |
|  | Central - Temporal | 0.535 ± 0.085 | 0.513 ± 0.099 | 6162.5 | -0.166 | 0.027 | 0.124 | ns |
|  | Frontal - Central | 0.550 ± 0.081 | 0.540 ± 0.092 | 6690.5 | -0.095 | 0.207 | 0.377 | ns |
|  | Frontal - Frontal | 0.618 ± 0.109 | 0.637 ± 0.116 | 8101.5 | 0.096 | 0.202 | 0.377 | ns |
|  | Frontal - Occipital | 0.699 ± 0.123 | 0.744 ± 0.151 | 8717.5 | 0.179 | 0.017 | 0.105 | ns |
|  | Frontal - Parietal | 0.553 ± 0.079 | 0.565 ± 0.101 | 8000.5 | 0.082 | 0.274 | 0.457 | ns |

Table S5(continued)

| **Frequency Band** | **Connection (Edge)** | **HC**  **(n=154, Mean ± SD)** | **ADS**  **(n=96, Mean ± SD)** | ***U*** | ***r*** | ***P*** | ***P*_FDR_** | **Sig.** |
| --- | --- | --- | --- | --- | --- | --- | --- | --- |
| Theta | Frontal - Temporal | 0.619 ± 0.106 | 0.605 ± 0.129 | 6442 | -0.129 | 0.088 | 0.227 | ns |
|  | Occipital - Occipital | 0.908 ± 0.198 | 0.980 ± 0.207 | 8813.5 | 0.192 | 0.011 | 0.1 | ns |
|  | Parietal - Occipital | 0.571 ± 0.119 | 0.612 ± 0.114 | 8999.5 | 0.217 | 0.004 | 0.077 | ns |
|  | Parietal - Parietal | 0.691 ± 0.140 | 0.675 ± 0.150 | 6956 | -0.059 | 0.434 | 0.619 | ns |
|  | Temporal - Occipital | 0.600 ± 0.118 | 0.635 ± 0.130 | 8466.5 | 0.145 | 0.053 | 0.189 | ns |
|  | Temporal - Parietal | 0.488 ± 0.089 | 0.484 ± 0.093 | 6998.5 | -0.053 | 0.48 | 0.64 | ns |
|  | Temporal - Temporal | 0.635 ± 0.100 | 0.600 ± 0.115 | 5988.5 | -0.19 | 0.012 | 0.1 | ns |
| Alpha | Central - Central | 0.595 ± 0.089 | 0.591 ± 0.117 | 7105.5 | -0.039 | 0.607 | 0.687 | ns |
|  | Central - Occipital | 0.653 ± 0.131 | 0.652 ± 0.134 | 7367.5 | -0.003 | 0.966 | 0.996 | ns |
|  | Central - Parietal | 0.537 ± 0.084 | 0.536 ± 0.093 | 7378.5 | -0.002 | 0.981 | 0.996 | ns |
|  | Central - Temporal | 0.578 ± 0.105 | 0.567 ± 0.100 | 6990.5 | -0.054 | 0.471 | 0.64 | ns |
|  | Frontal - Central | 0.645 ± 0.105 | 0.628 ± 0.116 | 6614.5 | -0.105 | 0.162 | 0.325 | ns |
|  | Frontal - Frontal | 0.696 ± 0.130 | 0.702 ± 0.122 | 7803.5 | 0.056 | 0.46 | 0.64 | ns |
|  | Frontal - Occipital | 0.717 ± 0.148 | 0.755 ± 0.166 | 8389.5 | 0.135 | 0.073 | 0.219 | ns |
|  | Frontal - Parietal | 0.595 ± 0.091 | 0.619 ± 0.109 | 8393.5 | 0.135 | 0.072 | 0.219 | ns |
|  | Frontal - Temporal | 0.737 ± 0.157 | 0.708 ± 0.133 | 6863.5 | -0.071 | 0.342 | 0.527 | ns |
|  | Occipital - Occipital | 0.841 ± 0.237 | 0.918 ± 0.256 | 8606.5 | 0.164 | 0.029 | 0.124 | ns |
|  | Parietal - Occipital | 0.546 ± 0.113 | 0.594 ± 0.122 | 9119.5 | 0.234 | 0.002 | 0.057 | ns |
|  | Parietal - Parietal | 0.646 ± 0.157 | 0.675 ± 0.160 | 8222 | 0.112 | 0.136 | 0.291 | ns |
|  | Temporal - Occipital | 0.612 ± 0.143 | 0.657 ± 0.156 | 8728.5 | 0.181 | 0.016 | 0.105 | ns |
|  | Temporal - Parietal | 0.520 ± 0.103 | 0.537 ± 0.109 | 8016.5 | 0.084 | 0.262 | 0.449 | ns |
|  | Temporal - Temporal | 0.713 ± 0.150 | 0.684 ± 0.129 | 6674.5 | -0.097 | 0.197 | 0.377 | ns |

Table S5(continued)

| **Frequency Band** | **Connection (Edge)** | **HC**  **(n=154, Mean ± SD)** | **ADS**  **(n=96, Mean ± SD)** | ***U*** | ***r*** | ***P*** | ***P*_FDR_** | **Sig.** |
| --- | --- | --- | --- | --- | --- | --- | --- | --- |
| Beta | Central - Central | 0.543 ± 0.083 | 0.523 ± 0.102 | 6468.5 | -0.125 | 0.097 | 0.233 | ns |
|  | Central - Occipital | 0.637 ± 0.118 | 0.635 ± 0.137 | 7066.5 | -0.044 | 0.559 | 0.648 | ns |
|  | Central - Parietal | 0.482 ± 0.070 | 0.476 ± 0.082 | 6905.5 | -0.066 | 0.382 | 0.573 | ns |
|  | Central - Temporal | 0.530 ± 0.088 | 0.510 ± 0.102 | 6381.5 | -0.137 | 0.069 | 0.219 | ns |
|  | Frontal - Central | 0.537 ± 0.077 | 0.526 ± 0.094 | 6723.5 | -0.09 | 0.23 | 0.405 | ns |
|  | Frontal - Frontal | 0.632 ± 0.117 | 0.643 ± 0.125 | 7767.5 | 0.051 | 0.5 | 0.644 | ns |
|  | Frontal - Occipital | 0.713 ± 0.131 | 0.751 ± 0.170 | 8231.5 | 0.114 | 0.131 | 0.291 | ns |
|  | Frontal - Parietal | 0.541 ± 0.088 | 0.557 ± 0.098 | 8170.5 | 0.105 | 0.162 | 0.325 | ns |
|  | Frontal - Temporal | 0.614 ± 0.114 | 0.606 ± 0.139 | 6927.5 | -0.063 | 0.404 | 0.591 | ns |
|  | Occipital - Occipital | 0.940 ± 0.221 | 1.020 ± 0.247 | 8713.5 | 0.179 | 0.018 | 0.105 | ns |
|  | Parietal - Occipital | 0.589 ± 0.118 | 0.634 ± 0.132 | 8842.5 | 0.196 | 0.009 | 0.1 | ns |
|  | Parietal - Parietal | 0.675 ± 0.138 | 0.681 ± 0.159 | 7432.5 | 0.005 | 0.943 | 0.992 | ns |
|  | Temporal - Occipital | 0.626 ± 0.131 | 0.664 ± 0.146 | 8545.5 | 0.156 | 0.038 | 0.143 | ns |
|  | Temporal - Parietal | 0.489 ± 0.093 | 0.491 ± 0.105 | 7322.5 | -0.009 | 0.901 | 0.983 | ns |
|  | Temporal - Temporal | 0.624 ± 0.104 | 0.609 ± 0.126 | 7035.5 | -0.048 | 0.522 | 0.648 | ns |

Note. Data are presented as Mean ± SD. HC = Healthy Controls; ADS = Adolescents with depressive symptoms; Metrics denote the dynamic functional connectivity features between specific cortical regions;*U* = Mann-Whitney U statistic; *r* = effect size; *P* = uncorrected P-value; *P*_FDR_= P-value corrected using the False Discovery Rate method. * indicates *P*_FDR_ < 0.05, ** indicates *P*_FDR_ < 0.01, *** indicates *P*_FDR_< 0.001; ns = not significant.

Table S6. Inter-group differences in mesoscale higher-order topological features (TDA) between the HC and ADS groups across four frequency bands

| **Frequency Band** | **Region** | **HC**  **(n=154, Mean ± SD)** | **ADS**  **(n=96, Mean ± SD)** | ***U*** | ***r*** | ***P*** | ***P*_FDR_** | **Sig.** |
| --- | --- | --- | --- | --- | --- | --- | --- | --- |
| Delta | Central | 0.430 ± 0.613 | 0.339 ± 0.533 | 7078.5 | -0.042 | 0.566 | 0.774 | ns |
|  | Frontal | 0.373 ± 0.494 | 0.304 ± 0.455 | 7028.5 | -0.049 | 0.504 | 0.774 | ns |
|  | Occipital | 0.520 ± 0.828 | 0.415 ± 0.591 | 7581 | 0.026 | 0.72 | 0.8 | ns |
|  | Parietal | 0.377 ± 0.530 | 0.355 ± 0.491 | 7439 | 0.006 | 0.932 | 0.932 | ns |
|  | Temporal | 0.447 ± 0.654 | 0.438 ± 0.606 | 7692 | 0.041 | 0.58 | 0.774 | ns |
| Theta | Central | 0.444 ± 0.595 | 0.306 ± 0.472 | 6323.5 | -0.145 | 0.05 | 0.2 | ns |
|  | Frontal | 0.336 ± 0.468 | 0.231 ± 0.429 | 6192.5 | -0.162 | 0.027 | 0.2 | ns |
|  | Occipital | 0.620 ± 1.003 | 0.300 ± 0.485 | 6345.5 | -0.142 | 0.048 | 0.2 | ns |
|  | Parietal | 0.372 ± 0.520 | 0.267 ± 0.483 | 6065 | -0.18 | 0.014 | 0.2 | ns |
|  | Temporal | 0.416 ± 0.591 | 0.249 ± 0.410 | 6216 | -0.159 | 0.03 | 0.2 | ns |
| Alpha | Central | 0.319 ± 0.359 | 0.419 ± 0.450 | 8348 | 0.129 | 0.085 | 0.283 | ns |
|  | Frontal | 0.320 ± 0.389 | 0.404 ± 0.450 | 8216 | 0.111 | 0.137 | 0.391 | ns |
|  | Occipital | 0.706 ± 1.043 | 0.706 ± 1.057 | 7594 | 0.027 | 0.715 | 0.8 | ns |
|  | Parietal | 0.453 ± 0.514 | 0.507 ± 0.534 | 7905.5 | 0.069 | 0.356 | 0.711 | ns |
|  | Temporal | 0.300 ± 0.382 | 0.354 ± 0.369 | 8098.5 | 0.096 | 0.202 | 0.506 | ns |
| Beta | Central | 0.450 ± 0.540 | 0.452 ± 0.513 | 7700 | 0.042 | 0.579 | 0.774 | ns |
|  | Frontal | 0.387 ± 0.495 | 0.337 ± 0.357 | 7827.5 | 0.059 | 0.431 | 0.774 | ns |
|  | Occipital | 0.553 ± 0.707 | 0.441 ± 0.639 | 6758 | -0.086 | 0.244 | 0.542 | ns |
|  | Parietal | 0.476 ± 0.573 | 0.486 ± 0.574 | 7612.5 | 0.03 | 0.69 | 0.8 | ns |
|  | Temporal | 0.452 ± 0.542 | 0.420 ± 0.522 | 7340 | -0.007 | 0.926 | 0.932 | ns |

Note. Data are presented as mean ± SD. HC = healthy controls; ADS = adolescents with depressive symptoms. Values represent region-averaged normalized node persistence (Meso-NP), calculated by averaging node persistence across electrodes within each anatomical region. *U* = Mann–Whitney U statistic; *r* = effect size; *P* = uncorrected P-value; *P*_FDR_ = P-value corrected using the false discovery rate method. * indicates *P*_FDR_ < 0.05, ** indicates *P*_FDR_ < 0.01, *** indicates *P*_FDR_< 0.001; ns = not significant.

Table S7. Significant inter-group differences in microscale static functional connectivity (sFC) between the HC and ADS groups across four frequency bands

| **Frequency Band** | **Connection (Edge)** | **HC**  **(n=154, Mean ± SD)** | **ADS**  **(n=96, Mean ± SD)** | ***U*** | ***r*** | ***P*** | ***P*_FDR_** | **Sig.** |
| --- | --- | --- | --- | --- | --- | --- | --- | --- |
| Theta | Fp1 - FCz | 0.179 ± 0.027 | 0.198 ± 0.043 | 9640.5 | 0.304 | <0.001 | 0.046 | * |
|  | Fz - FCz | 0.179 ± 0.029 | 0.205 ± 0.063 | 9725.5 | 0.316 | <0.001 | 0.046 | * |

Note. Data are presented as Mean ± SD. HC = Healthy Controls; ADS = Adolescents with depressive symptoms; Metrics denote the microscale static phase-lag coupling strength between specific electrode nodes. Due to the massive number of microscale features, this table only lists connections with statistically significant differences (*P*_FDR_ < 0.05). *U* = Mann-Whitney U statistic; *r* = effect size; *P* = uncorrected P-value; *P*_FDR_= P-value corrected using the False Discovery Rate method. * indicates *P*_FDR_ < 0.05, ** indicates *P*_FDR_ < 0.01, *** indicates *P*_FDR_< 0.001; ns = not significant.

Table S8. Significant inter-group differences in microscale dynamic functional connectivity (dFC) between the HC and ADS groups across four frequency bands

| **Frequency Band** | **Connection (Edge)** | **HC**  **(n=154, Mean ± SD)** | **ADS**  **(n=96, Mean ± SD)** | ***U*** | ***r*** | ***P*** | ***P*_FDR_** | **Sig.** |
| --- | --- | --- | --- | --- | --- | --- | --- | --- |
| Theta | F4 - Oz | 1.116 ± 0.215 | 1.238 ± 0.171 | 9776.5 | 0.323 | <0.001 | 0.031 | * |
|  | Fp2 - Oz | 1.104 ± 0.220 | 1.229 ± 0.186 | 9671.5 | 0.308 | <0.001 | 0.036 | * |

Note. Data are presented as Mean ± SD. HC = Healthy Controls; ADS = Adolescents with depressive symptoms; Metrics denote the microscale dynamic functional connectivity features between specific electrode nodes. Due to the massive number of microscale features, this table only lists connections with statistically significant differences (*P*_FDR_ < 0.05). *U* = Mann-Whitney U statistic; *r* = effect size; *P* = uncorrected P-value; *P*_FDR_= P-value corrected using the False Discovery Rate method. * indicates *P*_FDR_ < 0.05, ** indicates *P*_FDR_ < 0.01, *** indicates *P*_FDR_< 0.001; ns = not significant.

Table S9. Significant inter-group differences in microscale higher-order topological nodal features between the HC and ADS groups

| **Frequency Band** | **Dimension** | **Electrode Node** | **HC**  **(n=154, Mean ± SD)** | **ADS**  **(n=96, Mean ± SD)** | ***U*** | ***r*** | ***P*** | ***P*_FDR_** | **Sig.** |
| --- | --- | --- | --- | --- | --- | --- | --- | --- | --- |
| Theta | NF | Fp2 | 0.688 ± 1.297 | 0.312 ± 0.768 | 5898 | -0.202 | 0.001 | 0.048 | * |
|  | NF | F4 | 0.948 ± 1.639 | 0.469 ± 1.145 | 5890 | -0.203 | 0.001 | 0.048 | * |
|  | NP | F4 | 0.425 ± 0.770 | 0.129 ± 0.334 | 5712.5 | -0.227 | <0.001 | 0.032 | * |
|  | NP | Fp2 | 0.266 ± 0.493 | 0.121 ± 0.283 | 5923.5 | -0.199 | 0.001 | 0.048 | * |
| Alpha | NF | FT7 | 0.981 ± 1.661 | 1.521 ± 1.689 | 9271.5 | 0.254 | <0.001 | 0.032 | * |
|  | NP | FT7 | 0.298 ± 0.544 | 0.380 ± 0.508 | 9038.5 | 0.223 | 0.001 | 0.048 | * |

Note. Data are presented as Mean ± SD. HC = Healthy Controls (n = 154); ADS = Adolescents with depressive symptoms (n = 96); Dimension denotes the specific nodal topological property, where NF = Node Frequency and NP = Node Persistence; Electrode Node indicates the specific EEG channel. Due to the massive number of microscale features, this table only lists nodes with statistically significant differences (*P*_FDR_ < 0.05). *U* = Mann-Whitney U statistic; *r* = effect size; *P* = uncorrected P-value; *P*_FDR_= P-value corrected using the False Discovery Rate method. * indicates *P*_FDR_ < 0.05, ** indicates *P*_FDR_ < 0.01, *** indicates *P*_FDR_< 0.001; ns = not significant.

Table S10. Significant microscale multidimensional nodal features used for exploratory scalp-level convergence mapping between the HC and ADS groups

| **Frequency Band** | **Dimension** | **Electrode Node** | **HC**  **(n=154, Mean ± SD)** | **ADS**  **(n=96, Mean ± SD)** | ***U*** | ***r*** | ***P*** | ***P*_FDR_** | **Sig.** |
| --- | --- | --- | --- | --- | --- | --- | --- | --- | --- |
| Theta | NF | Fp2 | 0.688 ± 1.297 | 0.312 ± 0.768 | 5898 | -0.202 | 0.001 | 0.048 | * |
|  |  | F4 | 0.948 ± 1.639 | 0.469 ± 1.145 | 5890 | -0.203 | 0.001 | 0.048 | * |
|  | NP | F4 | 0.425 ± 0.770 | 0.129 ± 0.334 | 5712.5 | -0.227 | <0.001 | 0.032 | * |
|  |  | Fp2 | 0.266 ± 0.493 | 0.121 ± 0.283 | 5923.5 | -0.199 | 0.001 | 0.048 | * |
|  | dFC | Oz | 1.125 ± 0.183 | 1.205 ± 0.121 | 9339.5 | 0.263 | <0.001 | 0.032 | * |
|  | sFC | FCz | 0.181 ± 0.015 | 0.191 ± 0.023 | 9823.5 | 0.329 | <0.001 | 0.006 | ** |
|  |  | P3 | 0.177 ± 0.017 | 0.190 ± 0.029 | 9550.5 | 0.292 | <0.001 | 0.025 | * |
|  |  | Fz | 0.181 ± 0.016 | 0.190 ± 0.026 | 9388.5 | 0.270 | <0.001 | 0.032 | * |
|  |  | CPz | 0.179 ± 0.017 | 0.187 ± 0.022 | 9441.5 | 0.277 | <0.001 | 0.032 | * |
|  |  | F3 | 0.180 ± 0.013 | 0.191 ± 0.028 | 9257.5 | 0.252 | <0.001 | 0.046 | * |
|  |  | C4 | 0.179 ± 0.014 | 0.190 ± 0.028 | 9244.5 | 0.251 | <0.001 | 0.046 | * |
|  |  | Fp1 | 0.180 ± 0.014 | 0.190 ± 0.025 | 9201.5 | 0.245 | 0.001 | 0.048 | * |
|  |  | FC3 | 0.180 ± 0.015 | 0.192 ± 0.033 | 9173.5 | 0.241 | 0.001 | 0.048 | * |
| Alpha | NF | FT7 | 0.981 ± 1.661 | 1.521 ± 1.689 | 9271.5 | 0.254 | <0.001 | 0.032 | * |
|  | NP | FT7 | 0.298 ± 0.544 | 0.380 ± 0.508 | 9038.5 | 0.223 | 0.001 | 0.048 | * |

Note. Data are presented as mean ± SD. HC = healthy controls (n = 154); ADS = adolescents with depressive symptoms (n = 96). Dimension denotes the nodal feature index, including nodal static connectivity strength (sFC), nodal dynamic connectivity variability (dFC), node persistence (NP), and node frequency (NF). Electrode Node indicates the corresponding EEG channel. This table summarizes significant nodal features used for exploratory scalp-level convergence mapping, and only nodes showing FDR-corrected group differences are listed (*P*_FDR_ < 0.05). *U* = Mann-Whitney U statistic; *r* = effect size; *P* = uncorrected P-value; *P*_FDR_= P-value corrected using the False Discovery Rate method. * indicates *P*_FDR_ < 0.05, ** indicates *P*_FDR_ < 0.01, *** indicates *P*_FDR_< 0.001; ns = not significant.

Table S11. Reference-Independent Sensitivity Analysis of Theta-Band Static Functional Connectivity Using Fixed Proportional Thresholds

| **Fixed Sparsity** | **Metric** | **HC**  **(n=154, Mean ± SD)** | **ADS**  **(n=96, Mean ± SD)** | ***U*** | ***r*** | ***P*** | ***P*_FDR_** | **Sig.** |
| --- | --- | --- | --- | --- | --- | --- | --- | --- |
| Top 15% | Theta sFC | 0.511 ± 0.021 | 0.532 ± 0.052 | 9404.5 | -0.272 | 0.0003 | 0.0003 | *** |
| Top 20% | Theta sFC | 0.483 ± 0.019 | 0.504 ± 0.052 | 9479.5 | -0.282 | 0.0002 | 0.0003 | *** |
| Top 25% | Theta sFC | 0.458 ± 0.018 | 0.479 ± 0.052 | 9538.5 | -0.29 | 0.0001 | 0.0003 | *** |

Note. Data are presented as Mean ± SD. HC = Healthy Controls (n = 154); ADS = Adolescents with depressive symptoms (n = 96). This sensitivity analysis evaluated the robustness of the macroscopic findings by forcing all brain networks to maintain identical fixed topological sparsities (retaining the top 15%, 20%, and 25% of strongest connections), completely independent of the healthy reference group's baseline. Theta sFC denotes the static functional connectivity in the Theta band. Non-parametric Mann-Whitney U tests were applied for intergroup comparisons. U = Mann-Whitney U statistic; r = rank-biserial correlation effect size; P = uncorrected P-value; PFDR = P-value corrected using the False Discovery Rate method.* indicates *P*_FDR_ < 0.05, ** indicates *P*_FDR_ < 0.01, *** indicates *P*_FDR_< 0.001; ns = not significant

Table S12. Targeted Absolute SD Validation of Initially Significant Dynamic Functional Connectivity Pathways Across Meso- and Micro-Scales.

| **Scale** | **Frequency Band** | **Connection (Edge)** | **HC**  **(n=154, Mean ± SD)** | **ADS**  **(n=96, Mean ± SD)** | **U** | **r** | **P** | ***P*_FDR_** | **Sig.** |
| --- | --- | --- | --- | --- | --- | --- | --- | --- | --- |
| Micro | Theta | F4 - Oz | 0.21153 ± 0.02263 | 0.22181 ± 0.02391 | 8859.5 | 0.199 | 0.0083 | 0.0083 | ** |
| Micro | Theta | Fp2 - Oz | 0.21011 ± 0.02285 | 0.22076 ± 0.02380 | 8868.5 | 0.2 | 0.0079 | 0.0083 | ** |
| Meso | Delta | Frontal - Frontal | 0.13486 ± 0.02211 | 0.14342 ± 0.02282 | 9284.5 | 0.256 | <0.001 | 0.002 | ** |

Note. Data are presented as Mean ± SD. HC = Healthy Controls (n = 154); ADS = Adolescents with depressive symptoms (n = 96); U = Mann-Whitney U statistic; r = rank-biserial correlation effect size; P = uncorrected P-value; P_FDR = P-value corrected using the False Discovery Rate method. To explicitly rule out the potential mean-driven arithmetic artifacts in the Temporal CV metric, the absolute temporal standard deviation (without mean normalization) was extracted for supplementary validation. Statistically, the FDR correction was strictly designed as a targeted post-hoc analysis restricted only to these initially significant pathways (n=3) to confirm genuine dynamic destabilization, circumventing redundant whole-brain multiple comparisons.* indicates *P*_FDR_ < 0.05, ** indicates *P*_FDR_ < 0.01, *** indicates *P*_FDR_< 0.001; ns = not significant

Table S13. Node-Level Stability Probabilities of Most Significant Connections (MSCs) Derived from Bootstrap Resampling

| **Electrode** | **Stability Probability (%)** | **Electrode** | **Stability Probability (%)** | **Electrode** | **Stability Probability (%)** |
| --- | --- | --- | --- | --- | --- |
| P8 | 100.00% | Fz | 95.00% | Fp1 | 83.60% |
| T7 | 99.70% | TP7 | 94.90% | CP3 | 83.50% |
| P7 | 99.30% | C3 | 94.90% | Pz | 81.70% |
| O2 | 99.00% | F8 | 93.70% | Oz | 81.20% |
| FT8 | 98.80% | C4 | 93.50% | O1 | 80.00% |
| FC4 | 97.70% | FT7 | 92.80% | F3 | 79.00% |
| FCz | 97.10% | F7 | 92.20% | T8 | 65.80% |
| TP8 | 95.30% | FC3 | 88.10% | CPz | 62.90% |
| CP4 | 95.20% | Cz | 85.20% | F4 | 24.60% |
| P3 | 95.10% | P4 | 84.10% | Fp2 | 0.00% |

Note. The table presents the stability probability for all 30 whole-brain EEG electrode nodes, sorted in descending order. Stability probability is defined as the percentage of times a specific node was successfully extracted as part of the Most Significant Connections (MSCs) across 1,000 independent bootstrap resampling iterations. The MSC extraction pipeline was strictly based on the eigenvector centrality of the static functional connectivity (sFC) matrices.

Table S14.Non-parametric permutation and bootstrapping validation for initially significant macroscopic network features

| **Analysis Level** | **Family Size (N)** | **Frequency Band** | **Metrics** | **Effect Size (r)** | **Bootstrap 95% CI** | **Original *P*_FDR_** | **Permutation *P*_FDR_** | **Perm_Sig.** |
| --- | --- | --- | --- | --- | --- | --- | --- | --- |
| Macro | 4 | Theta | sFC | 0.291 | [0.150, 0.429] | 0.000 *** | 0.0003 | *** |
| Macro | 4 | Theta | Fractional Time S1 | -0.178 | [-0.323, -0.024] | 0.035 * | 0.0356 | * |
| Macro | 4 | Beta | Fractional Time S1 | -0.222 | [-0.361, -0.075] | 0.012 * | 0.012 | * |
| Macro | 4 | Beta | Fractional Time S3 | 0.21 | [0.067, 0.348] | 0.013 * | 0.0125 | * |
| Macro | 4 | Delta | Total Transitions | -0.164 | [-0.311, -0.014] | 0.030 * | 0.0289 | * |
| Macro | 4 | Theta | Total Transitions | -0.167 | [-0.315, -0.016] | 0.030 * | 0.0289 | * |
| Macro | 4 | Alpha | Total Transitions | -0.235 | [-0.376, -0.086] | 0.007 ** | 0.0073 | ** |
| Macro | 4 | Beta | Total Transitions | -0.177 | [-0.325, -0.028] | 0.030 * | 0.0289 | * |
| Macro | 4 | Beta | Transition S2 → S1 | -0.235 | [-0.376, -0.089] | 0.007 ** | 0.0068 | ** |
| Macro | 4 | Beta | Transition S2 → S2 | 0.209 | [0.063, 0.351] | 0.022 * | 0.0218 | * |
| Macro | 4 | Beta | Transition S2 → S3 | 0.172 | [0.049, 0.293] | 0.020 * | 0.0178 | * |
| Macro | 4 | Beta | Transition S3 → S3 | 0.098 | [0.019, 0.180] | 0.043 * | 0.041 | * |

Note: To robustly filter out potential false positives in high-dimensional testing and account for cross-feature correlations, a family-wise non-parametric permutation test (100,000 iterations) strictly preserving the original family sizes was applied. Original *P*_FDR_ = the initial P-value corrected by intra-level False Discovery Rate; Bootstrap 95% CI = the 95% confidence intervals of the effect size derived from a 5,000-iteration Bootstrapping procedure; Permutation *P*_FDR_ = the updated P-value after the family-wise permutation penalty. Features maintaining a Permutation *P*_FDR_ < 0.05 with CIs completely excluding zero are considered robustly significant. * indicates Permutation *P*_FDR_ < 0.05, ** indicates Permutation *P*_FDR_ < 0.01, *** indicates Permutation P_FDR < 0.001; ns = not significant (trend-level effect).

Table S15. Non-parametric permutation and bootstrapping validation for initially significant mesoscopic network features

| **Analysis Level** | **Family Size (N)** | **Frequency Band** | **Metrics** | **Effect Size (r)** | **Bootstrap 95% CI** | **Original P_FDR** | **Permutation P_FDR** | **Perm_Sig.** |
| --- | --- | --- | --- | --- | --- | --- | --- | --- |
| Meso_sFC | 60 | Theta | Frontal-Frontal | 0.253 | [0.109, 0.396] | 0.012 * | 0.0125 | * |
| Meso_sFC | 60 | Theta | Frontal-Central | 0.314 | [0.180, 0.453] | 0.002 ** | 0.0015 | ** |
| Meso_sFC | 60 | Theta | Central-Central | 0.296 | [0.149, 0.438] | 0.002 ** | 0.0015 | ** |
| Meso_sFC | 60 | Theta | Central-Temporal | 0.235 | [0.093, 0.374] | 0.018 * | 0.0166 | * |
| Meso_sFC | 60 | Theta | Central-Parietal | 0.281 | [0.135, 0.414] | 0.004 ** | 0.003 | ** |
| Meso_sFC | 60 | Theta | Temporal-Parietal | 0.23 | [0.077, 0.384] | 0.019 * | 0.0174 | * |
| Meso_sFC | 60 | Theta | Parietal-Parietal | 0.206 | [0.058, 0.347] | 0.044 * | 0.0417 | * |
| Meso_sFC | 60 | Alpha | Parietal-Occipital | -0.204 | [-0.346, -0.056] | 0.044 * | 0.0417 | * |
| Meso_sFC | 60 | Alpha | Occipital-Occipital | -0.235 | [-0.372, -0.091] | 0.018 * | 0.0166 | * |
| Meso_sFC | 60 | Beta | Central-Temporal | 0.2 | [0.055, 0.348] | 0.046 * | 0.0488 | * |
| Meso_dFC | 60 | Delta | Frontal-Frontal | 0.278 | [0.129, 0.426] | 0.013 * | 0.012 | * |

Note: To robustly filter out potential false positives in high-dimensional testing and account for cross-feature correlations, a family-wise non-parametric permutation test (100,000 iterations) strictly preserving the original family sizes was applied. Original *P*_FDR_ = the initial P-value corrected by intra-level False Discovery Rate; Bootstrap 95% CI = the 95% confidence intervals of the effect size derived from a 5,000-iteration Bootstrapping procedure; Permutation *P*_FDR_ = the updated P-value after the family-wise permutation penalty. Features maintaining a Permutation *P*_FDR_ < 0.05 with CIs completely excluding zero are considered robustly significant. * indicates Permutation *P*_FDR_ < 0.05, ** indicates Permutation *P*_FDR_ < 0.01, *** indicates Permutation *P*_FDR_ < 0.001; ns = not significant (trend-level effect).

Table S16. Non-parametric permutation and bootstrapping validation for initially significant microscopic network features

| **Analysis Level** | **Family Size (N)** | **Frequency Band** | **Metrics** | **Effect Size (r)** | **Bootstrap 95% CI** | **Original P_FDR** | **Permutation P_FDR** | **Perm_Sig.** |
| --- | --- | --- | --- | --- | --- | --- | --- | --- |
| Micro_sFC | 1740 | Theta | sFC (Fp1–FCz) | 0.304 | [0.160, 0.446] | 0.046 * | 0.0783 | ns |
| Micro_sFC | 1740 | Theta | sFC (Fz–FCz) | 0.316 | [0.169, 0.454] | 0.046 * | 0.0174 | * |
| Micro_dFC | 1740 | Theta | dFC (Fp2–Oz) | 0.308 | [0.164, 0.448] | 0.036 * | 0.0348 | * |
| Micro_dFC | 1740 | Theta | dFC (F4–Oz) | 0.323 | [0.185, 0.459] | 0.031 * | 0.0348 | * |
| Micro_Nodal | 480 | Theta | Nodal sFC (Fp1) | 0.245 | [0.093, 0.392] | 0.048 * | 0.0454 | * |
| Micro_Nodal | 480 | Theta | Nodal sFC (F3) | 0.252 | [0.105, 0.397] | 0.046 * | 0.0432 | * |
| Micro_Nodal | 480 | Theta | Nodal sFC (Fz) | 0.27 | [0.126, 0.411] | 0.032 * | 0.0261 | * |
| Micro_Nodal | 480 | Theta | Nodal sFC (C4) | 0.251 | [0.101, 0.399] | 0.046 * | 0.0432 | * |
| Micro_Nodal | 480 | Theta | Nodal sFC (P3) | 0.292 | [0.149, 0.433] | 0.025 * | 0.0261 | * |
| Micro_Nodal | 480 | Theta | Nodal sFC (FC3) | 0.241 | [0.094, 0.385] | 0.048 * | 0.0454 | * |
| Micro_Nodal | 480 | Theta | Nodal sFC (CPz) | 0.277 | [0.132, 0.418] | 0.032 * | 0.0261 | * |
| Micro_Nodal | 480 | Theta | Nodal sFC (FCz) | 0.329 | [0.187, 0.467] | 0.006 ** | 0.0048 | ** |
| Micro_Nodal | 480 | Theta | Nodal dFC (Oz) | 0.263 | [0.119, 0.401] | 0.032 * | 0.0261 | * |
| Micro_Nodal | 480 | Theta | Nodal NP (Fp2) | -0.199 | [-0.313, -0.082] | 0.048 * | 0.0454 | * |
| Micro_Nodal | 480 | Theta | Nodal NP (F4) | -0.227 | [-0.342, -0.111] | 0.032 * | 0.0261 | * |
| Micro_Nodal | 480 | Theta | Nodal NF (Fp2) | -0.202 | [-0.316, -0.086] | 0.048 * | 0.0446 | * |
| Micro_Nodal | 480 | Theta | Nodal NF (F4) | -0.203 | [-0.318, -0.085] | 0.048 * | 0.0454 | * |
| Micro_Nodal | 480 | Alpha | Nodal NP (FT7) | 0.223 | [0.083, 0.361] | 0.048 * | 0.0454 | * |
| Micro_Nodal | 480 | Alpha | Nodal NF (FT7) | 0.254 | [0.119, 0.383] | 0.032 * | 0.0261 | * |

Note: To robustly filter out potential false positives in high-dimensional testing and account for cross-feature correlations, a family-wise non-parametric permutation test (100,000 iterations) strictly preserving the original family sizes was applied. Original *P*_FDR_ = the initial P-value corrected by intra-level False Discovery Rate; Bootstrap 95% CI = the 95% confidence intervals of the effect size derived from a 5,000-iteration Bootstrapping procedure; Permutation *P*_FDR_ = the updated P-value after the family-wise permutation penalty. Features maintaining a Permutation *P*_FDR_ < 0.05 with CIs completely excluding zero are considered robustly significant. * indicates Permutation *P*_FDR_ < 0.05,** indicates Permutation *P*_FDR_ < 0.01, *** indicates Permutation *P*_FDR_ < 0.001; ns = not significant (trend-level effect).

Table S17. Quality-Control Metrics for Retained Clean EEG Data After Preprocessing

| **Group** | **N** | **Retained clean EEG duration, s** | **Retained 2-s clean epochs** | **Retained proportion** | **Rejected proportion** |
| --- | --- | --- | --- | --- | --- |
| HC | 154 | 342.86 ± 11.42 | 171.43 ± 5.71 | 0.952 ± 0.032 | 0.048 ± 0.032 |
| ADS | 96 | 340.75 ± 13.28 | 170.38 ± 6.64 | 0.946 ± 0.037 | 0.054 ± 0.037 |

Note. Data are presented as mean ± SD. Retained clean EEG duration indicates the length of artifact-free EEG data retained after preprocessing. Retained 2-s clean epochs were calculated by segmenting the retained artifact-free EEG data into non-overlapping 2-s epochs. Retained proportion was calculated relative to the original 360-s resting-state EEG recording, and rejected proportion was calculated as 1 minus the retained proportion. HC = healthy controls; ADS = adolescents with depressive symptoms; EEG = electroencephalography.

Supplementary

Table S18. Targeted dependence check between node frequency and node persistence at theta-band Fp2 and F4

| **Frequency band** | **Node** | **Feature pair** | **Sample** | **N** | **Spearman’s ρ** | **P value** |
| --- | --- | --- | --- | --- | --- | --- |
| Theta | Fp2 | NF vs. NP | All participants | 250 | 0.981 | <0.001 |
| Theta | F4 | NF vs. NP | All participants | 250 | 0.980 | <0.001 |

Note. NF = node frequency; NP = node persistence. Spearman correlation analysis was performed as a targeted post-hoc dependence check because NF and NP were both derived from the same H₁ persistent-homology computation. The high correlations indicate that concurrent NF and NP findings at Fp2 and F4 should be interpreted as convergent alterations across related topological indices rather than as evidence for two independent abnormal dimensions.
